# Carbon Dots as an Emergent Class of Sustainable Antifungal Agents

**DOI:** 10.1021/acsnano.5c03934

**Published:** 2025-07-02

**Authors:** Mattia Ghirardello, Javier Ramos-Soriano, M. Carmen Galan

**Affiliations:** † Department of Chemistry and Instituto de Investigación en Química de la Universidad de La Rioja (IQUR), Universidad de La Rioja, Logroño 26006, Spain; ‡ Institute for Biocomputation and Physics of Complex Systems (BIFI), University of Zaragoza, Zaragoza 50018, Spain; § Instituto de Investigaciones Químicas (IIQ), 16379 CSIC - Universidad de Sevilla, Av. Américo Vespucio 49, Seville 41092, Spain; ∥ School of Chemistry, 1980 University of Bristol, Cantock’s Close BS8 1TS, United Kingdom

**Keywords:** carbon dot nanomaterials, fluorescent probes, antifungal, bioimaging probes, carbon dot composites, nanomaterials, theranostics, diagnostics

## Abstract

Carbon-based functional nanomaterials with distinct photoluminescent properties have gained significant attention for their diverse applications in bioimaging, biomedicine, and antimicrobial treatments. Among these, carbon dots (CDs) have emerged as promising fluorescent nanomaterials due to their inherent photoluminescence properties, high stability, water solubility, ease of functionalization, biocompatibility, and low synthetic cost. Many strategies have been developed for their synthesis, utilizing a myriad of carbon precursors from small molecules to bulk or waste materials, which influence their structural and photoluminescent properties. Their fluorescence emission and functionality can be tuned through heteroatom doping, surface modifications, and reaction conditions, making them highly tunable nanomaterials suitable for applications in sensing, catalysis, anticancer and antimicrobial treatments, and biomedical imaging. This review explores various types of synthesized CDs, their structural features, and their applications in fungal bioimaging, antifungal therapies, and protective food packaging to demonstrate their potential in combating fungal resistance and contamination challenges.

## Graphical abstract

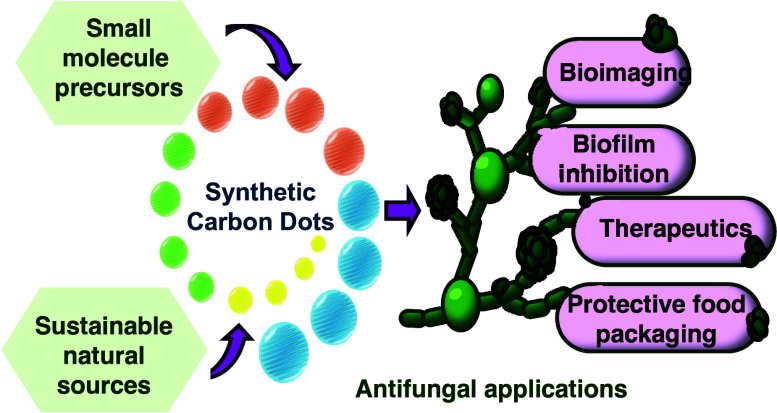

## 1 Introduction to Synthetic Carbon Dots: General Structural Features and Applications

Carbon-based functional nanomaterials with distinctive photoluminescent properties have garnered significant attention as valuable synthetic platforms that have found applications in a wide range of bioimaging and biological and biomedical applications^1–3^ due to their remarkable chemical and photochemical stability, simplicity of preparation, high water solubility, ease of functionalization, biocompatibility, and low synthetic cost.^4,5^ Among those, carbon dots (CDs), which are quasi-spherical nanomaterials under 10 nm of particle size and share several attributes of semiconductor inorganic quantum dots (QDs) such as broadband excitation spectra^6^ and tunable fluorescence,^7^ but without the associated cytotoxicity, have emerged as promising fluorescent platforms for a myriad of diverse applications.^8^ Since the serendipitous discovery of CDs in 2004 by Xu et al.^9^ during the purification of single-walled carbon nanotubes generated from arc-discharge soot, a variety of top-down and bottom-up synthetic protocols to generate CDs with distinct luminescent and physicochemical properties have been reported.^10,11^ CDs can be synthesized from a range of carbon-containing precursors such as small molecules, polymers, and biomass.^12^ In top-down approaches, large-sized (bulk) carbon materials, that already feature aromatic motifs within their structures, such as carbon nanotubes, graphite, graphene, or candle soot, are subjected to laser ablation, oxidative cleavage, hydrothermal, solvochemical, microwave, or ultrasonic-assisted processes to generate fluorescent nanoparticles.^13–15^ The crystalline makeup of top-down derived CDs is usually highly sp^2^ in character, which is transferred from the sp^2^-enriched starting materials. It is also possible to obtain CDs from green as sustainable sources lacking polyaromatic motifs such as industrial waste, biomass (e.g., plant, fungi, and bacteria extracts), which can be decomposed under thermal conditions to undergo dehydration and carbonization events leading to a polyaromatization process, and eventually the formation of the CD core. Conversely, bottom-up approaches rely on small molecules (e.g., sugars, amino acids, citric acid, etc.) or polymers as carbon precursors, which undergo thermal decomposition through chemical or hydrothermal oxidation, microwave, acid-mediated reflux, ultrasonic irradiation or silica nanoparticle-templated synthetic processes to seed the formation of CDs. Typically, N-, S-, P-, or B-containing doping agents are introduced during the reaction to enhance/tune the photoluminescent properties of the materials (Figure 1).^2,5,14,15^

**Figure 1:**
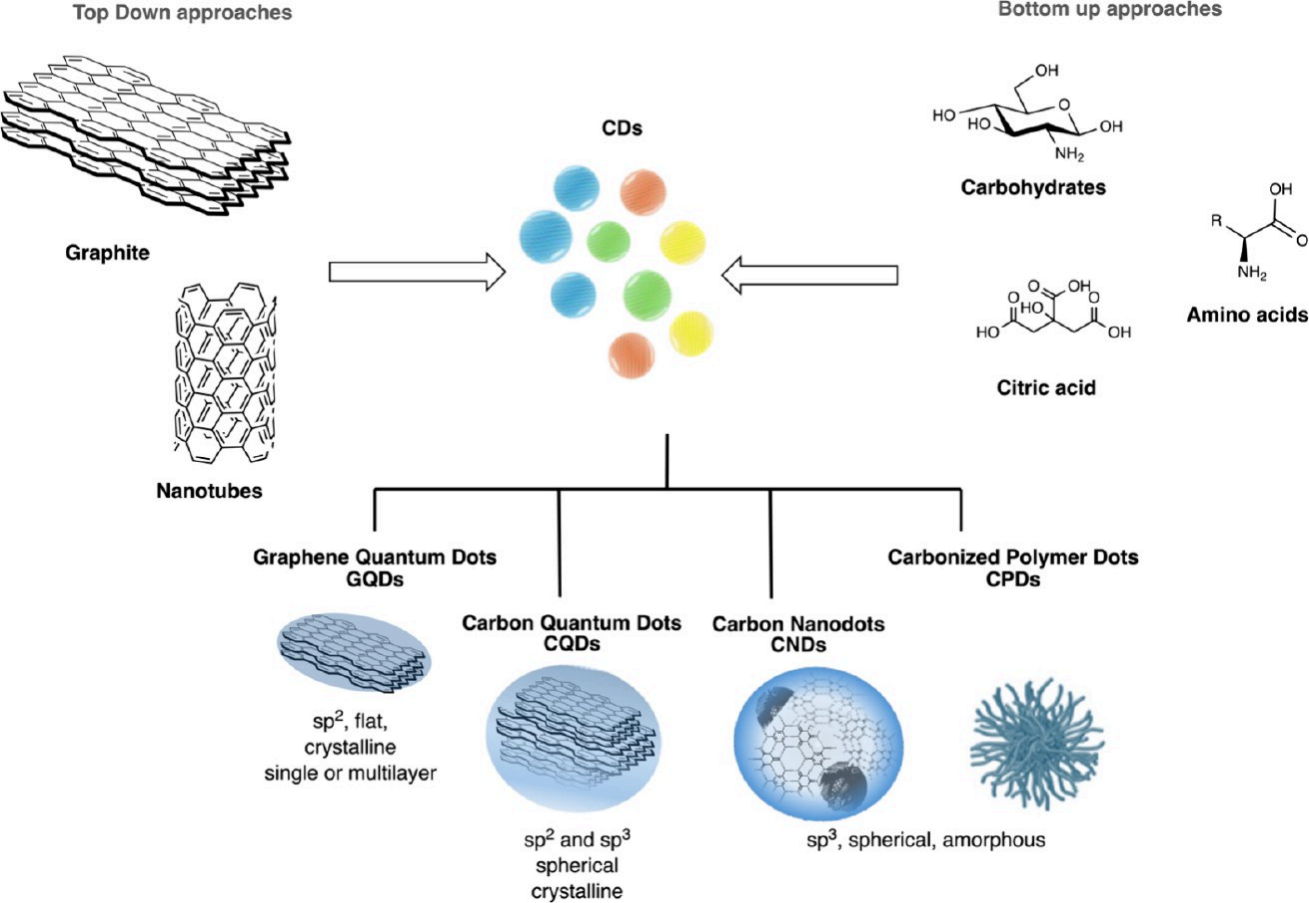
Schematic representation of top-down and bottom-up synthetic approaches for the preparation of different CDs.

The use of chemically defined starting materials minimizes batch-to-batch variability of the CDs, when compared to approaches that employ natural sources or feedstocks. CDs obtained through the latter methods are generally less sp^2^ crystalline and exhibit more amorphous morphologies. It is important to note that no two CD preparations produce identical nanoparticles, as variations in the ratio and composition of starting materials, additives, solvent, temperature, reaction vessel type, and other parameters influence the final molecular composition and structure of the CDs. Consequently, even minor adjustments to the synthesis process can lead to distinct properties.^16^ Thus, using defined bottom-up approaches ensures greater control over the synthetic process, enhancing the reproducibility and consistency of the nanomaterials.

CDs can be classified into four categories based on their composition: graphene quantum dots, carbon quantum dots, carbon nanodots, and carbonized polymer dots.^17^ Typically, graphene quantum dots have an anisotropic structure made of 2D layered graphene connected by π conjugation and chemical groups present at the edges or in the interlayer defects.^14,17,18^ Whereas carbon quantum dots have a spherical crystal core, carbon nanodots have an amorphous core and carbonized polymer dots are cross-linked or aggregated polymers (Figure 1).^17–19^

The chemical stability, fluorescence quantum efficiencies, and emission profile of CDs are dictated by the structural and morphological properties of the nanomaterials. Early studies established that high fluorescence quantum yields (FQYs) can be attributed to the restricted intramolecular motion of fluorophores within a rigid core structure of CDs.^20^ Other reports have suggested that high FQYs are associated with nitrogen-enriched chemical groups which introduce trap N-states and facilitate the radiative recombination.^21^ Moreover, the surface functionalization of CDs also regulates their fluorescence absorption and emission wavelength,^22^ since these groups can introduce additional manifold of states (midgap states) below the conduction band.^23,24^ Most CDs emit blue or green fluorescence and heteroatoms such as N, P, and S among others are incorporated within the CD structure to enhance the fluorescent properties of these nanomaterials, increasing the fluorescent quantum yield and influencing the absorbance and emission band typically toward a blue or in some cases a red shift.^25^ Indeed, red and NIR emissive CDs have also been reported in recent years,^2^ which are ideal for biomedical applications and can be generated by introducing codopant reagents during the synthetic process, for example, by the incorporation of electron-acceptor moieties rich in sulfoxide/carbonyl groups bound to the outer layers to help promote radiative relaxations in the red spectral region^24^ or by increasing graphitic N within the structure.^26^ Furthermore, CDs can be excited by light energy which generates a charge separation and the formation of electrons and hole pairs trapped on the CD’s irregular surface, generating an excited state that decays via fluorescent emission to promote reactive oxygen species (ROS) formation. Moreover, the chemical structure of the CD surface, including the presence of reactive functional groups, such as amino (−NH_2_), carboxyl (−CO_2_H), and hydroxy (−OH) groups, is also determined by the choice of precursor materials. Whether derived from organic small molecules such as amino acids, citric acid, or biomass-based sources, these precursors play a crucial role in shaping the structural and chemical properties of the nanomaterial and its interactions with the environment. Several lines of evidence indicate that multiple factors coexist and affect the emission and functionality of the resulting nanoparticles.^27^ Despite recent progress, there are still gaps in our current fundamental understanding of CD fluorescence modulation, and the exact origin and set of parameters that correlate chemical structure with fluorescence emission have been elusive, due to the heterogeneity within the CD structures. As a result, many synthetic efforts are still a result of trial and error. However, there is now a common agreement that the fluorescence properties are linked to CD size, surface defects, functional groups, and oxidation state.^22,28^ Nonetheless, owing to their photoluminescence tuneability, stability, biocompatibility, and large surface area, which allows for the attachment of targeting molecules and biomolecules, applications across many fields such as catalysis,^29^ sensing,^30^ antimicrobial,^31^ antifungal,^32^ and antiviral^33^ agents, gene delivery,^34^ cell imaging,^35^ *in vitro* theranostics,^36^ photosynthesis augmentation,^37^ food preservation,^38^ cancer sensing,^39^ and photocatalysis,^40^ among others, have been realized.^41^

Synthetic methods for accessing CDs and their anticancer and antimicrobial applications have been extensively reviewed^2,5,10,25,42^ and as such will not be covered in extensive detail within this perspective. A recent article has reviewed the use of CDs as bioactive antifungal agents with special focus on mode of action and potential applications.^32^ In this review, we aim to provide an up to date overview of the different types of synthetic approaches and surface modifications that generate bioactive CDs and their use in fungi bioimaging and detection, as antifungal agents, and as component of protective food packaging, which is an emergent area of research.

## 2 Fungal Infections and Current Challenges

The increasing prevalence of fungal resistance and invasive fungal infections poses a significant global health threat, requiring innovative solutions.^43^ Millions of people develop life-threatening invasive fungal infections, nearly half of whom will die despite the availability of antifungal treatments.^44^ These infections result in more deaths annually than tuberculosis or malaria.^45^ The incidence of fungal infections has risen due to factors such as increased use of immunosuppressive and invasive medical procedures as well as the global HIV/AIDS epidemic. In these cases, opportunistic pathogens like *Candida* species, *Cryptococcus neoformans*, and *Aspergillus fumigatus* take advantage of weakened defenses. Fungal infections can also affect immunocompetent individuals who have suffered physical trauma, which disrupts natural protective barriers and significantly increases susceptibility to infection. Additionally, fungal diseases also cause significant nonlethal health burdens, including asthma, allergies, chronic and often disfiguring skin infections, and keratitis, a condition that can lead to blindness.^44^

The emergence of multi-drug-resistant pathogens has further exacerbated the above challenges, highlighting the urgent need for alternative therapeutic strategies that provide high efficacy with minimal toxicity and environmental impact.^32^ The most prominent example is given by the indiscriminate use of azoles, widely used as antifungal agents in medicine and agriculture, contaminate soil and water, promoting antifungal resistance.^46^ Persistent in the environment, they exert selective pressure on fungi, fostering resistant strains such as *A. fumigatus*. This resistance threatens human health and crop protection.^47^

Numerous studies have demonstrated the efficacy of CDs in eradicating a broad range of pathogens, including fungi, by targeting cellular structures and disrupting metabolic processes. CDs have been effectively used as antifungal agents and in preventing biofilm formation. Beyond clinical applications, such as bioimaging and theranostic approaches that combine drug delivery and intracellular diagnostics, these nanomaterials have shown great potential in agriculture for controlling fungal pathogens in crops and food preservation by inhibiting fungal spoilage. Although the precise mechanisms underlying fungal eradication by CDs have yet to be fully elucidated, several consistent pathways have been identified in multiple studies. Those have been recently reviewed^32^ and will not be discussed in detail in this perspective. Instead, the next sections highlight recent synthetic developments in this area and applications.

## 3 Small-Molecule-Derived CDs and Applications

### 3.1 Fungi Labeling and Imaging Applications

The rapid and precise diagnosis of fungal infections remains a challenge. Among the many strategies available, direct bioimaging using fluorophores to label the fungal cell surface offer many advantages.^48^ Fungal eukaryotic cells possess a rigid membrane that provides structural support and protection against environmental stresses. This membrane is a dynamic barrier primarily composed of glycoproteins modified with N- and O-linked carbohydrates, glucans, and chitin,^49^ further complicating diagnostic efforts.

Notably, CD technology has demonstrated advanced capabilities for imaging applications. To that end, CDs of different compositions have been developed for fungal cell labeling applications (Table 1). Citric acid is one of the most widely used carbon sources, which is often combined with various dopants such alkyl and aromatic amines,^50^ boric acid,^51^ polyethylene glycol,^52^ or silyl derivatives.^53^ These combinations have yielded fluorescent fungal cell markers through various methods, including microwave-assisted, solvothermal, and low-temperature synthetic processes. In addition to citric acid, other carbon sources such as ascorbic acid, glycerol, and fluorescent dyes have been explored for producing multicolor fluorescent CDs suitable for bioimaging applications. The nanomaterials generated through these processes exhibited high quantum yields and excellent chemical stability, ideal for fungal cell labeling. These CDs are generally nontoxic to fungal cells, allowing the imaging and monitoring of live microorganisms, with minimal interference with the cellular metabolism, via fluorescence microscopy.^54^ Moreover, some CDs have been shown to selectively differentiate between live and dead fungal cells through a straightforward fluorescent staining procedure. The first of such example was reported by Tian et al.^51^ in 2021. The group synthesized CDs through a microwave hydrothermal method using citric acid as the carbon source which were codoped with boron and nitrogen. The CDs effectively labeled yeast cells within 1 min, enabling the rapid identification of live and dead cells based on fluorescence intensity around the cell, which was attributed to the staining of the overflown cellular material from the dead cells. Different studies also proved that CDs could be used to specifically stain dead fungal cells such as yeast,^55–57^ *P. italicum*,^55^ and *C. albicans.* ^58^ In two different studies, the Wu group synthesized green fluorescent sulfur doped CDs via a one-step hydrothermal method using rose bengal as carbon source and 1,4-dimercaptobenzene^59^ (Figure 2a) or cysteine^60^ as starting materials to provide the sulfur dopant. The cysteine-based CDs gave green-fluorescent nanoparticles (FQY of 78%) with an average size of 3.7 nm, displaying a net negatively charged surface with a zeta potential of −28 mV. The presence of the sulfur dopant was confirmed through Fourier transform infrared spectroscopy (FTIR) and X-ray photoelectron spectroscopy (XPS) through the identification of characteristic peaks (C–S, S–O_*x*_) demonstrating that no sulfhydryl group was present on the CDs surface due to oxidation into sulfates during the hydrothermal synthesis. These Cys-based nanomaterials were successfully used for the staining of three types of fungal cells including *C. albicans*, *T. reesei*, and yeast, concomitantly demonstrating high bioavailability toward a set of mammalian cells. Similarly, the 1,4-dimercaptobenzene-based CDs showed an average size of 1.6 nm (Figure 2b) and were used for the selective labeling of dead *C. albicans* cells which showed clear sign of membrane damage (Figure 2c) after 1 min incubation with the CDs (Figure 2e). Conversely, no fluorescent signal was present in live cells, allowing the rapid and selective discrimination of live cells over dead fungal cells (Figure 2d). Remarkably, the selective staining of dead cells proved to be effective with other species including bacterial (*E. coli* and *S. aureus*), fungal (*S. cerevisiae* and *T. reesei*), and mammalian (HPAEpiC and A549) cell lines. In agreement with other studies, the authors hypothesized that the selectivity of the fluorescent labeling was due to the CDs diffusion into the dead cells through the damaged cell surface. It is likely that electrostatic interactions between the positively charged amino groups present on the CD surface and the negatively charged intracellular components (e.g., nucleic acids) facilitate the specific staining of dead cells.^61^

**Figure 2:**
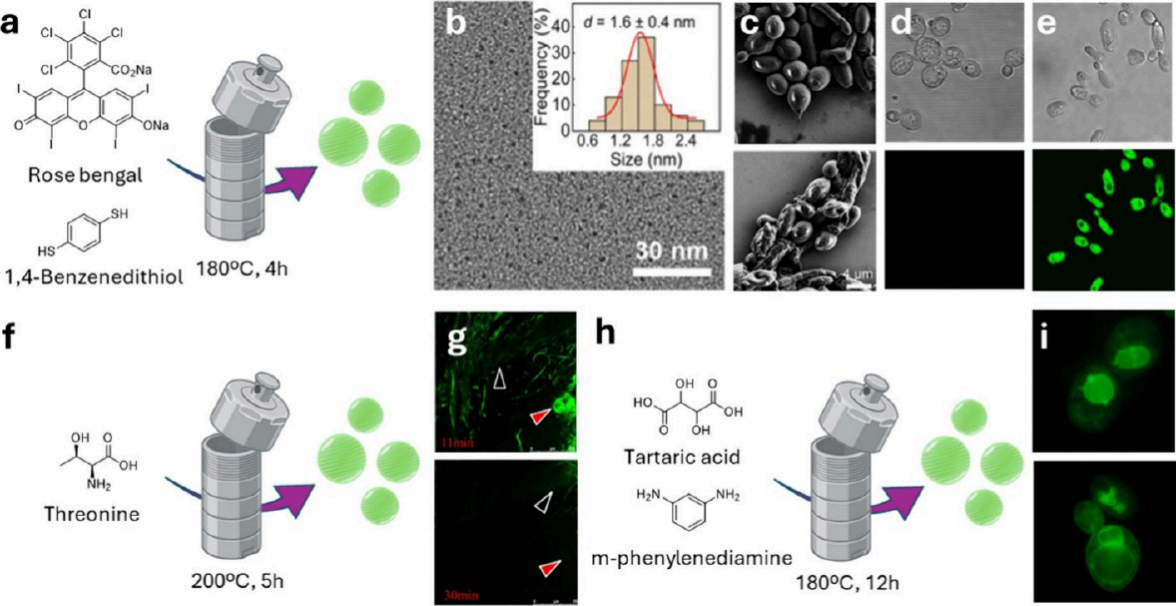
(a) Graphical representation of the CDs’ synthetic approach. (b) TEM image of the CDs. Insert: corresponding size distribution result. (c) SEM images of live (top) and dead *C. albicans* (bottom). (d) Bright field (top) and confocal fluorescence microscopic image (bottom) of CDs treated (20 μg/mL) live *C. albicans* cells. (e) Bright field (top) and confocal fluorescence microscopic image (bottom) of CDs treated (20 μg/mL) dead *C. albicans* cells. Images (a)–(e) have been adapted with permission from Yu et al.^59^ (f) Graphical representation of the CDs’ synthetic approach from Jin et al.^62^ (g) Representative confocal fluorescence images of *V. mali* in the infected apple tissue after 11 min (top) and 30 min (bottom) of incubation with basic PBS solution (pH = 10.0). Arrowhead with black cores demonstrate living fungal cells. Arrowhead with red cores indicates the infected apple tissue. (h) Graphical representation of the CDs’ synthetic approach adapted from Sun et al.^63^ (i) Fluorescence imaging of *C. albicans* stained with CDs localized in the nuclei and vacuoles. Images (g) and (i) have been adapted with permission from Jin et al.^62^ and Sun et al.^63^

**Table 1:** Summary of Small Molecule Derived-CDs and Their Applications

carbon source	doping agent	synthetic method	fungal species	application	ref.
methyl red	EDA	hydrothermal	*C. gloeosporioides*	bioimaging	^50a^
citric acid	acrylamide	solvothermal (DMF)	*F. oxysporum*	bioimaging	^50b^
glucose and citric acid	polyethylenimine	hydrothermal	*S. cerevisiae*	antifungal	^50c^
*m*-phenylene-diamine	-	hydrothermal	*C. albicans*, *S. cerevisiae*	bioimaging	^50d^
citric acid	EDA and boric acid	hydrothermal	*S. cerevisiae*	bioimaging	^51^
citric acid	polyethylene glycol	microwave	*C. albicans*	bioimaging	^52^
trisodium citrate	*N*-[3-(trimethoxy-silyl)propyl]ethylenediamine	hydrothermal	*P. ostreatus*	bioimaging	^53^
citric acid	l-cysteine	microwave	*S. cerevisiae*	bioimaging	^54^
erythrosin B	3-aminopropyl trimethoxysilane	hydrothermal	*S. cerevisiae*, *P. italicum*	bioimaging	^55–57^
citric acid	monoethanolamine	solvothermal (DMSO)	*A. flavus*, *A. fumigatus*	bioimaging	^55–57^
*o*-phenylene-diamine	urea	solvothermal (DMF)	*S. cerevisiae*	bioimaging	^55–57^
guanosine	-	hydrothermal	*C. albicans*	antifungal	^58^
rose bengal	1,4-dimercapto-benzene	hydrothermal	*C. albicans*	bioimaging	^59^
rose bengal	d,l-cysteine	hydrothermal	*C. albicans*	bioimaging	^60^
protamine sulfate	-	microwave	*S. cerevisiae*	bioavailability	^61a^
carbon nitride	-	sonication	*R. solani*	bioimaging, antifungal	^61b^
threonine	-	hydrothermal	*V. mali*	bioimaging	^62^
tartaric acid	*m*-phenylene-diamine or *p*-phenylenediamine	solvothermal (acetone) or hydrothermal	*C. albicans*	bioimaging	^63^
citric acid or 1,4-butane-diamine or *m*-phenylene-diamine	EDA or PEG-1500	microwave or hydrothermal	*P. adipose*	bioimaging, pH sensitive	^64^
l-asparagine	EDA	microwave	*C. gloeosporioides*	bioimaging, antifungal	^65^
tetracycline	EDA	hydrothermal	*S. cerevisiae*	bioimaging	^66a^
l-glutamic acid	EDA	microwave	*C.gloeosporioides*	bioimaging	^66b^
2-methoxy-1,4-naphthoquinone	-	solvothermal (DMSO)	*P. italicum*	antifungal	^67^
2-methoxy-1,4-naphthoquinone	-	solvothermal (DMSO)	*P. digitatum*	antifungal	^68^
commercially available	-	-	*P. infestans*	antifungal	^69^
citric acid	EDA	solvothermal (DMF)	*P. chrysosporium*	bioimaging, antifungal	^70^
commercially available	-	-	*V. dahliae*	antifungal	^71^
glucosamine hydrochloride	TTDDA	microwave	*P. capsici*	antifungal	^72^
citric acid	-	pyrolysis	*F. graminearum*	antifungal	^73^
d-glucosamine or citric acid or glucose	*m*-diaminobenzene or urea or polyacrylate sodium	microwave	*C. albicans*	biofilm inhibition	^75^
citric acid	EDA	solvothermal (formamide)	*C. albicans*	bioimaging, antifungal, biofilm inhibition	^76^
citric acid	-	pyrolysis	*S. cerevisiae*	antifungal	^77^
choline chloride and citric acid	urea	hydrothermal	*C. albicans*	antifungal	^78^
iopromide	EDA	hydrothermal	*C. albicans*	antifungal	^79^
glucose	-	hydrothermal	*C. albicans*	bioimaging, antifungal	^80a^
glucose	boric acid or sodium persulfate or urea	hydrothermal	*A. fumigatus*	antifungal	^80b^
			*F. solani*		
			*P. citrinum*		
			*C. albicans*		
			*R. rubra*		
d- or l-cysteine	-	hydrothermal	*C. albicans*	bioimaging, antifungal	^81^
citric acid	urea	microwave	*M. indicus*	antifungal	^82^
			*C. albicans*		
			*A. flavus*		
			*A. fumigatus*		
			*A. niger*		
			*P. notatum*		
citric acid and l-glutathione	polyvinyl polyamine or PEG-400	solvothermal	*F. solani*	bioimaging, antifungal	^83^

A different fungal cell labeling application of CDs was reported in 2022 by Zhang et al.^56^ The team synthesized citric acid-based CDs that exhibited a concentration-dependent emission that could be exploited to distinguish between *A. flavus* and *A. fumigatus* species. It was found that CD accumulation inside the fungal cells led to CD aggregation that resulted in a red shift in the fluorescent emission profile. Taking advantage of the differences in CD uptake by the two organisms, in particular, the higher concentration of CDs internalized in *A. fumigatus* compared to *A. flavus*, a stronger fluorescent emission in the long wavelength region in *A. fumigatus* allowed the accurate discrimination of the two fungi.

CDs have surpassed, in some instances, the capabilities of standard fluorophores in fluorescent imaging by allowing the accurate visualization of intracellular processes. In 2015, Jin et al.^62^ developed pH sensitive CDs through the hydrothermal decomposition of threonine, which was used as the main carbon source (Figure 2f). High resolution transmission electron microscopy (HRTEM) imaging revealed that the CDs were mono dispersed nanocrystals of near spherical morphology with an average diameter of 8.2 nm and rich in anime groups, as demonstrated by FTIR spectroscopy. The fluorescence intensity of these CDs was shown to decrease with an increase of pH, and this feature allowed the monitoring of intracellular pH changes in *V. mali*, a common plant pathogen, by confocal microscopy (Figure 2g). In this example, live fungal cells showed a bright fluorescent signal due to intracellular acidic pH. Upon cell death, the pH increases causing the quenching of the fluorescent signal, which allowed the probing of the pH variation inside the fungal cell. Recently, Wang et al.^64^ reported another study of pH-responsive CDs which were obtained by conjugating a pH sensitive moiety (alanine blue) to citric acid-based CDs produced through pyrolysis. The negatively charged CDs were mixed with alanine blue using a PEG 400 polymer to cross-link the pH sensitive moiety to the CD surface through hydrothermal pyrolysis. Fluorescence microscopy revealed a decrease in emission for the CDs located in the cytoplasm, endoplasmic reticulum, and Golgi apparatus, which was attributed to a local pH shift toward basic conditions. Using this probe, the group demonstrated that *P. adipose* requires an internal pH in the range of 7.10–7.25 for the biosynthetic production of fungal polysaccharides, which are valuable bioactive compounds used in pharmaceutical applications.

CDs were also employed as fluorescent probes to investigate the effect of fungicides in the intracellular components in fungal cells by visualizing the integrity of the cell membrane^65^ and for the intracellular detection of Fe^3+^ and Al^3+^ ions.^66^ In this context, Sun et al.^63^ described the selective labeling of fungal organelles using four different types of CDs which were produced via both hydrothermal and solvothermal degradation of tartaric acid and m-/p-phenylenediamine (Figure 2h) under different conditions for each. The nanoparticle exhibited variable sizes from 2.9 up to 7.2 nm diameter as determined by transmission electron microscopy (TEM) and the surface of the nanomaterials displayed O-and N-rich functional groups, which was corroborated by FTIR and zeta potential measurement. These probes were able to selectively target different organelles inside mammalian, plant, and fungal cells, enabling the imaging and monitoring of intracellular processes. Specifically, the CDs were able to selectively label the nuclei and the vacuoles of *C. albicans* (Figure 2i) and *S. cerevisiae* cells, emphasizing the versatility and potential of CDs for advanced bioimaging applications across various species and organisms.

### 3.2 Antifungal Applications

The applications discussed so far have involved the use of CDs as labeling material for fluorescent staining purposes of fungal cells. Notably, CDs have also shown promising application as fungicidal agents, with applications spanning from agricultural production to healthcare protection. The tunable synthesis of CDs, just by changing the carbon sources and the doping agents, provides easy access to structurally different CDs with distinct and in many cases high fungicidal activity, great environmental compatibility, and negligible toxicity for mammals.

In the agricultural sector, CDs have been explored for their potential to control plant pathogens. For instance, the Huang group developed 2-methoxy-1,4-naphthoquinone-based CDs which were produced through solvothermal degradation of the starting materials in DMSO. By incorporating the quinone molecule during the formation of the highly soluble CD nanoparticles, the group aimed to improve the water solubility properties of 2-methoxy-1,4-naphthoquinone, which was previously found to possess inhibitory activity against *P. digitatum*. HRTEM imaging showed that the CDs had a graphitic core structure with an average diameter of 4 nm. The surface was rich of *O*-functional groups as demonstrated by FTIR and XPS analysis. More importantly, these CDs showed excellent inhibition activity against *P. italicum* ^67^ and *P. digitatum*,^68^ two common plant pathogens that causes severe losses in the production of citrous fruits. Scanning electron microscopy (SEM) cell imaging showed morphological distortions, including damaged cell walls and organelles, and combined transcriptomics and metabolomics analyses revealed impaired metabolism, highlighting the potential of these CDs as antifungal agents. Subsequent studies by Kostov et al.^69^ further highlighted the efficacy of commercially available CDs as antifungal agents against an array of different fungi. Although details of the composition were not provided, the authors reported that the CDs inhibited *P. infestans* mycelial growth at low concentrations, with complete inhibition at concentrations as low as 40 μg/mL. Conversely, CDs could also be used to exert the opposite effect on benignant fungi. In the study reported by Qie et al.,^70^ the authors showed that the tailored-design of citric acid-based CD nanoparticles produced through the solvothermal degradation of citric acid and ethylenediamine could boost the growth of the white rot fungus *P. chrysosporium*. TEM imaging of the CDs showed that the nanoparticles possessed an average diameter of about 5 nm with a graphitic core structure displaying hydroxyl, amine, and carboxyl functions on the CD surface, as demonstrated by IR and XPS analysis. This fungus was capable of degrading lignin, which is a process that holds significant industrial and ecological applications for the valorization of agricultural byproducts. The team reported that the CDs slightly stimulated fungal dry weight growth at 50–100 μg/mL during the early stages of cultivation without impairing the lignin degradation efficiency.

Additionally, CDs were successfully applied as carriers for drug delivery inside fungal cells. Yin et al.^71^ developed different types of salicylic acid-based nanoprotectants for plant disease control, particularly targeting cotton Verticillium wilt caused by *V. dahliae*. The authors used commercially available CDs loaded with salicylic acid (a natural plant protectant) via CD surface adsorption by simply mixing the two components followed by dialysis purification to remove the excess of acid. The functionalized nanoparticles showed an average diameter of 6 nm measured through TEM imaging and a zeta potential of −15.37 mV, demonstrating the presence of an overall negatively charged surface as a consequence of the salicylic acid loading. The CD–salicylic acid formulation enhanced the uptake of salicylic acid in plant tissues, which was ascribed to the nanoscale size and surface properties of CDs, facilitating better delivery into cells and showing improved fungicidal activity compared to salicylic acid alone. Nonetheless, it is worth mentioning that other formulations, in which the hydrophilic and lipophilic diblock polymer was used as carriers, performed better than CDs, in terms of fungicidal activity and plant uptake.

Wang et al.^72^ pioneered the use of CDs as a vector for the treatment of *P. capsici*, a fungus who causes blight on over 70 crops through the delivery of CesA3-/OSBP1-double-stranded RNA (dsRNA). The silencing of these genes impacts the expression of cellulose synthase 3 (CesA3), a key component of cell-wall building, and oxysterol binding protein 1 (OSBP1) a protein involved in oxysterol transportation, which play key roles in fungal cell membrane composition and are necessary for fungal cell development. The author used glucosamine-based CDs functionalized with a PEGDA 1000 to provide a positive charge on the CD surface, which is required to bind and stabilize the dsRNA polymers through electrostatic interactions and facilitates fungal cell membrane internalization of the dsRNA sequences. This study emphasizes the ability of CDs to act as carriers and stabilizing agents that help prevent dsRNA degradation. The nanomaterial allows the sdRNA folding into smaller adducts with a reduced global negative charge, which promotes dsRNA delivery into cells thus leading to protection against *P. capsici*. A similar strategy was reported in 2024 by Gyawali et al.^73^ The authors explored the use of surface-functionalized CDs as carriers for delivering dsRNA to suppress *F. graminearum*, a fungal pathogen responsible for fusarium head blight in wheat. The CDs were synthesized using citric acid through a pyrolysis process and further functionalized using branched polyethylenimine (PEI, Figure 3a). The average nanoparticle diameter shifted from 2.02–5.21 to 2.19–5.07 nm after PEI functionalization, as measured by TEM and zeta potential (−1.71 mV before and +59.1 mV after PEI functionalization) (Figures 3b–c). The CD’s cationic functionalization was exploited to electrostatically bind dsRNA facilitating its delivery into fungal cells (Figure 3a). Two fungal genes, MGV1 (related to cell wall formation and fertility) and RAS1 (involved in spore germination and growth), were selected as RNA interference targets, and exogenous spray applications were tested on wheat spikes to control Fusarium Head Blight symptoms. This approach allowed for an enhanced delivery of the genetic material showing better fungal growth inhibition compared to the application of naked dsRNA. These results showcase the applicability of CDs in delivering RNA-based treatments for various plant diseases and antifungal applications (Figures 3d–e).

**Figure 3:**
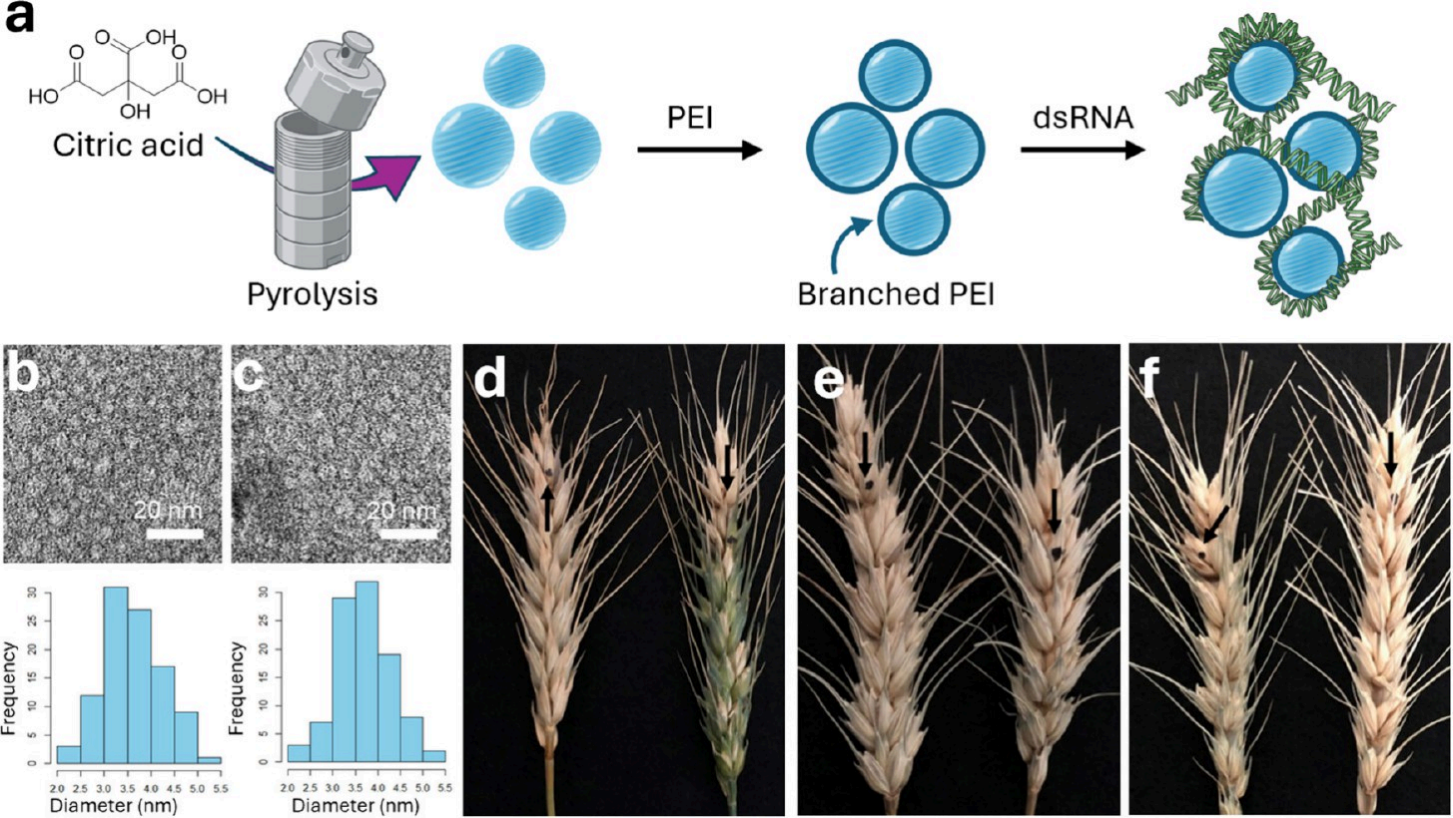
(a) Graphical representation of the CDs’ synthetic approach. (b) TEM images of CDs before PEI surface functionalization (top) and histograms depicting the size distribution of CDs (bottom). (c) TEM images of CDs after PEI surface functionalization (top) and histograms depicting the size distribution of CDs (bottom). (d) Restriction of infection symptoms in wheat heads after the application of naked dsRNA as well as dsRNA-CDs. (e) Control group using HT115 with empty vector. (f) H_2_O control. Images have been adapted with permission from Gyawali et al.^73^

### 3.3 Biofilm Inhibition Applications

Biofilms consist of microbial aggregates attached to a solid–liquid interface that are encased in a matrix of highly hydrated extracellular polymeric substances (EPSs). This matrix, composed primarily of polysaccharides, proteins, lipids, and nucleic acids, accounts for over 90% of the dry mass. Biofilm-associated infections are a major public health concern, as they reduce the efficacy and susceptibility of traditional antifungal drugs, particularly in the treatment of polymicrobial diseases.^74^

Functional groups can be engineered on the CD surface to target specific fungal processes such as biofilm formation, further enhancing the antifungal efficacy of the CDs. The fine-tuning of surface molecular features allows the development of CDs with tailored antifungal activity, minimizing toxicity to nontarget organisms. In their 2024 study, Sturabotti et al.^75^ investigated the antifungal potential of CDs against *C. albicans*, focusing on the effect of different molecular features on the nanomaterial’s surface. Three different types of CDs were synthesized by using a bottom-up approach under either microwave or autoclave heating conditions. The process allowed precise control over surface functional groups, for instance using d-glucosamine and *m*-diaminobenzene as the carbon sources positively charged amine rich CDs were generated. On the other hand, urea and citric acid were used for the synthesis of CO_2_H/NH_2_-rich negatively charged CDs, while d-glucose and polyacrylate sodium polymer were employed for the preparation of highly negatively charged CDs (Figure 4a). Superior efficiency in penetrating fungal cells was observed for the positively charged CDs (CDs-NH_2_). Moreover, the authors showed that these nanomaterials were able to inhibit cell adhesion and disrupted biofilm formation in *C. albicans* cell models at concentrations of 0.5 mg/mL (Figure 4b).

**Figure 4:**
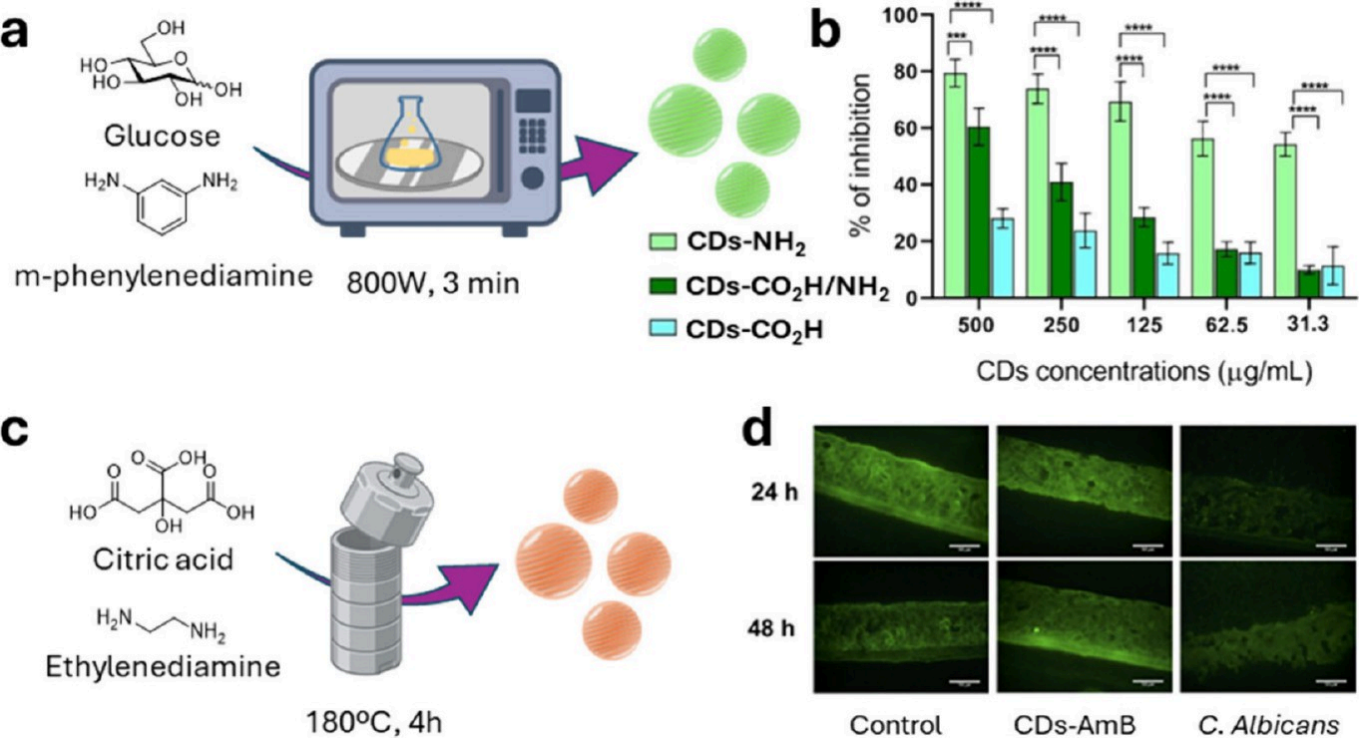
(a) Graphical representation of the CDs’ synthetic approach. (b) Inhibition of *C. albicans* adhesion to polystyrene wells in the presence of CDs–NH_2_ (lime green), CDs–CO_2_H/NH_2_ (green), and CDs–CO_2_H (blue) after 90 min. Images (a) and (b) have been adapted with permission from Sturabotti et al.^75^ (c) Graphical representation of the CDs’ synthetic approach. (d) Immunofluorescent staining (green representing E-cadherin) (scale bar is 50 μm) of a 3D reconstructed human oral epithelial model. E-cadherin is a calcium-dependent cell–cell adhesion molecule, and it plays an important role in mediating epithelial behavior and maintaining tissue integrity. One of the invasive approaches of *C. albicans* is to degrade E-cadherin by secreting lytic enzymes or manipulating the activity of epithelial cell calpain. The healthy control group showed similar behavior to the CDs-AmB treated tissues, while notable E-cadherin degradation is present in *C. albicans* treated tissues without CDs-AmB protectants. Images (c) and (d) have been adapted with permission from Li et al.^76^

The importance of the CD surface charge was also further documented by Li et al.^76^ in a study whereby red-emissive guanylated polyene-functionalized CDs were synthesized using a hydrothermal method with citric acid and ethylenediamine as core precursors (Figure 4c). The CD surface presented both carboxyl and amine groups that were used as orthogonal anchoring points for orthogonal functionalization with either guanidine as a cationic handle using 1H-pyrazole-1-carboxamidine hydrochloride as guanylation agent and the fungicidal drug Amphotericin B (AmB), via amide ligation between the carboxyl groups on the CD surface and the free amine moiety on AmB. The presence of a guanidine moiety on the CD surface enhanced the positive surface charges which helped boost the electrostatic interactions between the AmB-loaded nanoparticles and the negatively charged polysaccharides of *C. albican* cells/biofilms. The nanoparticles displayed effective antimicrobial and antibiofilm activity against *C. albicans* with no detectable toxicity to host cells, indicating their potential for safe topical applications. Moreover, in this study the CDs were able to penetrate and form a shielding layer within a 3D reconstructed human oral epithelial model. The team demonstrated that treatment with CDs-AmB prevented fungal invasion and protected the integrity of the epithelial tissues (Figure 4d). This protective mechanism indicates the potential for these CDs to act as a barrier in mucosal environments, reducing the risk of systemic infections.

### 3.4 Antifungal Applications in Animal Models

The unique physicochemical properties of CDs can provide targeted antifungal therapeutic approaches for biomedical applications. These materials can overcome the limitations of traditional treatments such as drug resistance and toxicity to neighboring cells by exerting a localized fungicidal activity that can be activated taking advantage of the photodynamic and photothermal properties of CDs. This versatility highlights the opportunities provided by CDs as novel platforms for addressing fungal infections and bridging the gap between sustainable and innovative healthcare solutions.

*C. albicans* represents the most studied fungal model since it is a common opportunistic pathogen residing in human microbiota. While typically harmless, it can cause infections ranging from superficial oral and vaginal candidiasis to severe systemic candidemia in immunocompromised individuals. Its ability to form biofilms and adapt to host environments complicates treatment, often leading to antifungal resistance and healthcare challenges.

Different reports have demonstrated the effective cytotoxicity of CDs against *C. albicans* using nanoparticles synthesized from different carbon precursors including citric acid,^77^ choline chloride,^78^ amino acids,^79^ and carbohydrate derivatives with different heteroatoms used as dopants.^80^ The general antimicrobial mechanism was attributed to irreversible cell membrane damage as a consequence of the combined photothermal and photodynamic activation of the CDs. For instance Song et al.^81^ reported the synthesis of d- or l-cysteine-based CDs prepared through thermal decomposition (Figure 5a). TEM analysis showed that the diameters of the chiral d- and l-CDs were 4.3 and 5.2 nm, respectively. These d- and l-CDs exhibited a FQY of 8.1% and 7.9%, respectively, and zeta potential measurement showed that both d- and l-CDs possessed net negatively charged surfaces of about −11.5 and −9.5 mV for l- and d-CDs, respectively. FTIR also showed similarities among the two types of CDs, featuring frequencies indicative of −OH, N–H, C–S, and carboxyl functional groups, among others, while XPS analysis confirmed the presence of C, N, S, and O groups on the surface of both types of CDs. It was found that the nanoparticles damaged the membrane of *C. albicans* and were able to induce a more extensive damage under dual 405/660 nm light irradiation (Figures 5b–c). ROS production was confirmed by flow cytometry using the fluorescent redox probe DCFH-DA. Furthermore, metabolic impairment in the ATP balance and leakage of nucleic acids confirmed that membrane damage and photodynamic activation were the main causes responsible for the antifungal activity of CDs.

**Figure 5:**
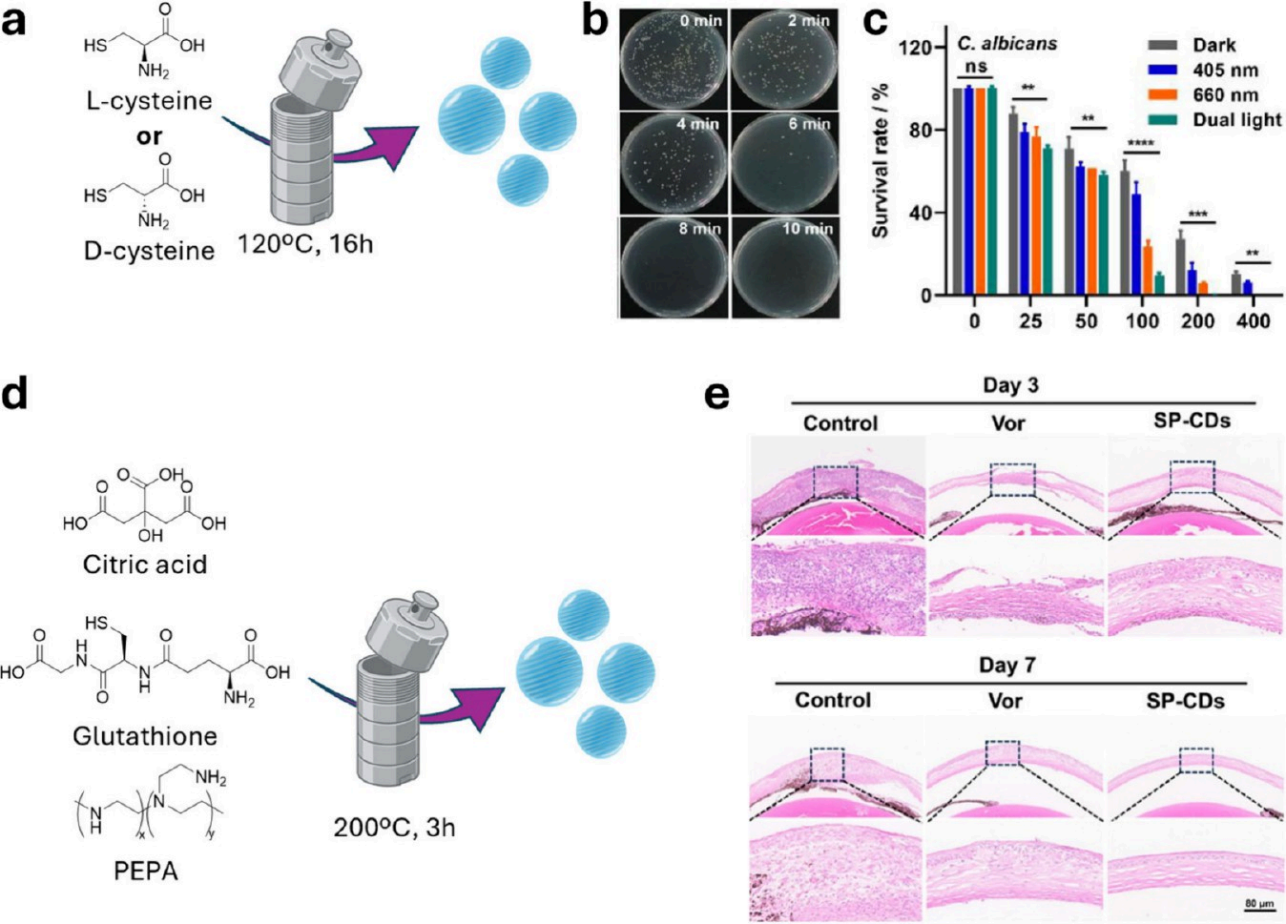
(a) Graphical representation of the CDs’ synthetic approach. (b) Plate pictures of *C. albicans* treated with CDs prepared from d-cysteine-CDs irradiated by a dual 405 nm and a 660 nm laser at 0.5 and 1.5 W cm^–2^, respectively, over 10 min. (c) Survival rates of *C. albicans* treated with CDs prepared from d-cysteine (0, 25, 50,100, 200, and 400 μg mL^–1^) under dark, irradiated by a 405 nm laser (0.5 W cm^–2^, 10 min), 660 nm (1.5 W cm^–2^, 10 min), and 405/660 nm (0.5 W cm^–2^, 1.5 W cm^–2^, 10 min) dual lasers, respectively. Images (a)–(c) have been adapted with permission from Song et al.^81^ (d) Graphical representation of the CDs’ synthetic approach. (e) Histological examination of mouse cornea tissue sections with hematoxylin and eosin (H·E) staining for different treatment groups and times (on days 3 and 7). Images (d) and (e) have been adapted with permission from Chen et al.^83^

*In vivo* applications have also confirmed the biocompatibility of CDs and their efficacy in eradicating fungal infections. Belal et al.^82^ utilized citric acid-based CDs produced through a microwave-assisted method to eradicate *M. indicus* infections. This fungus is a valuable microorganism used for food production and as a nutritional source; however, it may also cause joint infections which lead to arthritis. The authors showed that CDs were effective in inhibiting up to a 98% of *M. indicus* growth compared to other fungal species. Further *in vivo* studies of *M. indicus* infected skin lesions on rat models showed that topological ointment administration significantly reduced wound size and fungal load compared to the commercial cycloheximide antifungal treatment. The CD-based treatment led to faster wound contraction and healing, suggesting the applicability of CDs for *in vivo* applications.

Another example of antifungal CDs for *in vivo* applications comes from Chen et al.,^83^ who explored the use of ultrasmall positively charged CDs as a novel treatment for fungal keratitis, an eye infection originated by *F. solani* that can cause blindness. CDs with a 4.38 nm average diameter were synthesized from citric acid and glutathione in the presence of poly­(vinyl polyamine) (PEPA) through a solvent free stage-melting method (Figure 5d). Zeta potential analysis confirmed the presence of a net positive charge on the CD surface (+7.6 mV), and FTIR and XPS analysis confirmed the predominant presence of amine and hydroxy rich functional groups on the CD surface. The positively charged CDs were able to overcome the corneal barrier and effectively delivering antifungal properties to infected areas. Compared to traditional antifungal drugs such as voriconazole (Vor), the CDs showed enhanced fungal inhibition by disrupting fungal cell membranes and inducing oxidative stress with high biocompatibility and low host cell toxicity (Figure 5e). Investigations into the mode of action showed that the CD treatment temporarily increased the corneal cellular junctions, allowing the CD cellular uptake and permeation into the corneal barrier. *In vivo* tests on mice revealed superior therapeutic effects and recovery rates compared with conventional clinical treatments using the antifungal drug voriconazole. The lack of host cell toxicity and the great antimicrobial efficacy further highlights the potential of CDs in developing advanced nanomedicines for eye infections.

## 4 CDs from Sustainable Resources and Applications

### 4.1 Antifungal Applications

On account that CDs may retain or improve certain characteristics of their precursor properties in their final structure, another effective strategy for producing antifungal nanoagents involves using sustainable materials or biomass, as sources of carbon, for CD synthesis (Table 2). The protocols involve the use of microwave-assisted or hydrothermal methods, including autoclave,^84^ sand,^85^ or oil baths.^86^ An excellent example of CDs with enhanced characteristics compared to their source materials is exemplified by the work of Khan et al.^87^ This study explored the enhancement of antimicrobial properties, including antifungal effects, through the use of CDs derived from natural products, namely, the extract of the endophytic fungus *D. unshiuensis*. The authors synthesized blue-emitting, positively charged CDs, using a one-step solvothermal method. These CDs, which possessed amino, carboxy, hydroxy, and sulfite groups on the surface, exhibited significantly improved antimicrobial activity against fungi (as well as bacteria) compared to the original natural product extract, demonstrating low minimum inhibitory concentrations (MICs) of 18 and 24 μg/mL for *C. albicans* and *S. cerevisiae*, respectively. In a mouse model, CDs not only enhanced antimicrobial effects but also accelerated wound healing, all while maintaining good biocompatibility.

**Table 2:** Summary of Synthetic CDs from Sustainable Resources and Their Applications

carbon source	doping agent or composite	synthetic method	fungal species	application	ref.
*D. unshiuensis*	-	hydrothermal	*C. albicans*, *S. cerevisiae*	antifungal, biofilm inhibition	^87^
tamarind	-	hydrothermal	*C. albicans*	antifungal	^88,89^
seaweed	-	hydrothermal	*P. cubensis*	antifungal	^90^
pomegranate peels	-	microwave	*F. oxysporum*	antifungal	^91^
watermelon peels	-	microwave	*F. oxysporum*	antifungal	^91^
pumpkin seed kernel and seed shell	urea	microwave	*C. cladosporioides*	antifungal, bioimaging	^92^
forsythia	urea and ethanolamine	hydrothermal and microwave	*C. versicolor*	antifungal	^93^
chitosan and quaternary ammonium salt	urea and ethanolamine	hydrothermal and microwave	*C. versicolor*	antifungal, wood preservative	^94^
microcrystalline cellulose	-	hydrothermal and microwave	*C. albicans*	antifungal	^95^
fast-food packaging	H_2_O_2_	hydrothermal	*C. galbrata*, *C. tropicalis*	biofilm inhibition	^96^
Indian essential oils of clove, basil, turmeric, and cardamom	-	hydrothermal	-	food preservation	^86^
*V. nilotica* gum	chitosan/gelatin matrix	hydrothermal	-	food preservation	^97^
*Sophora japonica*	gelatin	hydrothermal	*B. cinerea*	antifungal, food preservation	^98^
baker’s yeast	nanocellulose membranes	hydrothermal	*A. flavus*	antifungal, food preservation	^99^
			*R. rubra*		
			*A. fumigatus*		
			*F. solani*		
pomelo peel	gelatin/alginate dialdehyde matrix	hydrothermal	*P. palitans*	antifungal, food preservation	^100^
			*A. fumigatus*		
lemon juice	-	hydrothermal	*B. cinerea*	antifungal, food preservation	^102^
onion juice	-	hydrothermal	*B. cinerea*	antifungal, food preservation	^102^
pomegranate juice	-	hydrothermal	*F. avenaceum*	bioimaging	^103^
papaya juice	-	hydrothermal	*A. aculeatus*	bioimaging	^104^
apple juice	-	hydrothermal	*M. oryzae*	bioimaging	^105^
*M. zapota*	sulfuric and phosphoric acids	hydrothermal	*A. aculeatus Fomitopsis* sp.	bioimaging	^106^
pea	-	hydrothermal	*C. neoformans*	tracking fungal infections	^107^
*S. aureus* or *E. coli* cells	-	hydrothermal	*S. cerevisiae*, *T. reesei*	bioimaging	^108^
*C. retusus* fruit	aqueous ammonia	hydrothermal	*C. albicans*, *C. neoformans*	bioimaging	^109^
*T. patula* flowers	-	hydrothermal	*M. oryzae*	bioimaging	^110^
Acacia concinna seeds	-	microwave	*Penicillium* sp.	bioimaging	^111^
plastic waste	-	hydrothermal	*L. taxodii*	bioimaging	^112^
*P. notoginseng*	-	hydrothermal	*S. cerevisiae*	bioimaging	^113^
cornstalks	-	hydrothermal	*C. albicans*	bioimaging	^114^
salmon DNA	-	hydrothermal	*C. albicans*	bioimaging	^115^
tender coconut water	-	microwave	*A. niger*	bioimaging	^118^
tomato pulp	urea and EDA	microwave	*C. gloeosporioides*, *V. mali*	bioimaging	^119^
			*B. berengeriana*		

Jhonsi et al.^88^ explored the antimicrobial activity of tamarind-derived CDs, demonstrating significant activity against *E. coli* (a bacterium) and *C. albicans* (a fungus) in comparison with other pathogenic organisms, with inhibition zones ranging from 7 to 12 mm. These CDs, which were obtained by a simple one pot hydrothermal method, are pH sensitive with negatively charged functional groups present on the surface, namely carboxylic acid, as determined by FTIR, XPS, and nuclear magnetic resonance (NMR).^89^ Due to the negatively charged CD surface, these CDs can interact with calf thymus DNA (ct-DNA) via intercalation.^88^ The proposed mechanism of action aligns with the broader research consensus that suggests that CDs can inhibit microbial growth through various mechanisms, including ROS generation and interactions via intercalation with microbial DNA.^31^ Taking advantage of the extensive π-conjugated structure and the abundance of −NH_2_ and −OH groups on the surface, alternative CDs generated from seaweed, which exhibited antifungal activity, were also employed for the loading of hydrophobic pesticides (flumorph) through a combination of hydrophobic interactions and hydrogen bonding.^90^ The authors hypothesized that the antifungal activity of CDs against *cucumber downy mildew* may be attributed to their oxygen-containing functional groups, which can adsorb onto the cell walls of bacteria and fungi before diffusing into them. These CDs are able to compromise the integrity of the cell membrane, ultimately causing cytoplasmic leakage.

CDs generated from the fast, eco-friendly, and cost-effective microwave synthesis method from waste materials, such as pomegranate and watermelon peels, have shown varying antifungal activity depending on the carbon sources.^91^ Although analyses using different techniques confirmed that both CDs share similar strong fluorescence, favorable size distribution (1–5 nm), and various key functional groups (−OH, −NH_2_, and −COOH) on their surface, only pomegranate-based CDs exhibited antifungal activity against the *F. oxysporum* strain. The authors could not provide a rational explanation for their results, other than that it is probably due to differences on the different molecular composition of the CDs, which is difficult to completely characterize. More recently, nitrogen-doped CDs were produced from pumpkin seed kernel, seed shell, and urea (as the nitrogen source), resulting in a 65.5% FQY.^92^ Characterization techniques confirmed the presence of heteroatom functional groups, including nitrogen, sulfur, phosphorus, potassium, magnesium, and zinc, on the surfaces of the graphitic carbon dots. These heteroatom-doped CDs exhibited significant antifungal activity against *C. cladosporioides*, a fungus responsible for economic losses in agricultural products. The presence of N, S, P, and Zn functional groups on the CDs’ surface contributes to their strong antifungal properties. Additionally, the inherent fluorescence (blue, cyan, green, and yellow-emitting colors, depending on the excitation wavelength) of these CDs enables enhanced bioimaging applications, allowing for effective visualization of biological systems.

Wang and co-workers synthesized nitrogen-doped CDs with natural antiwood-rot fungus activity using either Chinese herbal medicine-*Forsythia* ^93^ or the biobased material chitosan quaternary ammonium salt (HACC) from marine-derived chitosan,^94^ as the primary precursors. The synthesis also included urea and ethanolamine, which act as nitrogen dopants. While both microwave-assisted and hydrothermal strategies afforded strong blue-emissive fluorescent HACC-based CDs, the latter method produced CDs with superior antifungal properties against *C. versicolor* (a pathogenic fungus of wood), exhibiting a MIC of 1.8 mg/mL, significantly lower than that of HACC alone (40.0 mg/mL).^94^ A similar correlation when comparing CDs generated from microcrystalline cellulose using either hydrothermal versus microwave assisted methods and their respective antifungal properties (*C. albicans*) was previously observed in the literature, further suggesting that not just the starting materials, but also the type of carbonization protocol, have an effect in the ultimate molecular structure of the CDs.^95^

### 4.2 Biofilm Inhibition and Food Packaging Applications

Beyond antifungal applications, CDs from sustainable resources have also been effectively used in preventing fungal biofilm formation. The aforementioned CDs (Figure 6a) synthesized by Khan et al.^87^ inhibited biofilm formation of *C. albicans* fungus. Mechanistic studies revealed that CDs interact with microbial cell surfaces through electrostatic or hydrophobic interactions, penetrate within the cells, and distribute throughout the intracellular environment. This leads to cell membrane damage and disruption of the cell division cycle, ultimately causing microbial cell death (Figure 6b). A similar mechanism was proposed for CDs obtained from fast-food packaging, e.g., plastic plates and bowls.^96^ This approach of repurposing nonrecyclable plastic waste into valuable materials represents a crucial advancement in environmental conservation. These oxygen-rich CDs with graphite-like structure, inhibited biofilm formation of two different *Candida* strains, namely *C. galbrata* and *C. tropicalis*. At a concentration of 40 μg/mL, CDs reduced biofilm formation by ∼50% (Figure 6c). However, a significant reduction of over 90% was observed with a Minimal Biofilm Inhibitory Concentration (MBIC) of 100 μg/mL.

**Figure 6:**
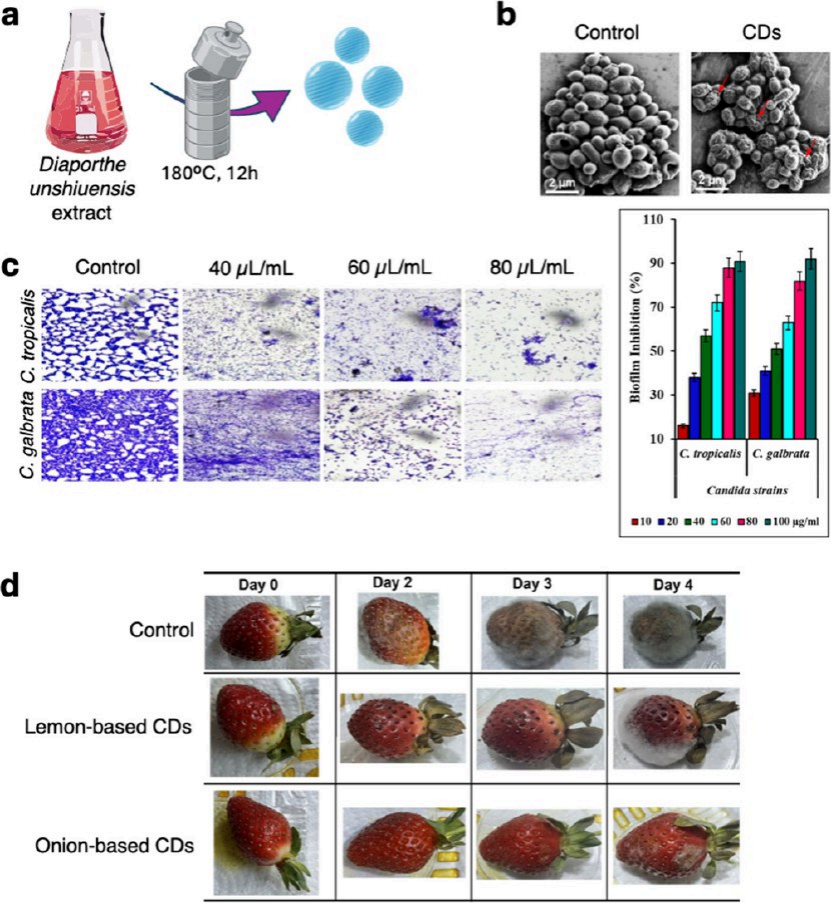
(a) Graphical representation of the CDs’ synthetic approach adapted from Khan et al.^87^ (b) SEM imaging of *C. albicans* before and after the incubation of CDs. Image adapted from Khan et al.^87^ (c) Inhibitory effects of biofilm formation (left) and antibiofilm properties (right) on CDs against *C. atropicalis* and *C. galbrata* strains. Image adapted from Thirumalaivasan et al.^96^ (d) The appearance of lemon-based and onion-based CD-packaged strawberries during the 4 days storage period. Image adapted with permission from Slewa et al.^102^

Moreover, this type of CDs from sustainable sources has also been used in food preservation for the inhibition of fungal spoilage. For example, CDs obtained from Indian essential oils of clove (*Eugenia caryophyllata*), basil (*Ocimum basilicum*), turmeric (*Curcuma longa*), and cardamom (*Elettaria cardamomum*) showed a favorable size distribution (well below 10 nm) and exhibited variable antifungal properties when tested on sliced bread.^86^

CDs derived from various carbon sources, including refined molecules and food byproducts, have also been successfully incorporated into gel and film formulations to create advanced functional materials with enhanced physicochemical properties. Numerous studies have highlighted that incorporating CDs into different matrices not only enhances the materials’ durability and UV-shielding capabilities but also enhances radical scavenging and enhances the antimicrobial properties of the finished film or coated products. These benefits have been effectively utilized in food storage and packaging applications. For instance, studies have demonstrated that perishable foods, such as fresh fruit, benefit significantly in terms of extended shelf life and increased microbial resistance when treated with CD-based films. This advancement holds significant commercial interest for the food industry, offering opportunities to extend the shelf life of perishable products and optimize the supply chain, such as transport and storage, by reducing food mold growth and enhancing UV protection. Improved UV shielding helps maintain stable storage temperatures and increases packaging efficiency, ultimately lowering logistics costs, enhancing food safety, and reducing food waste. The use of CDs derived from renewable sources further supports sustainability efforts, reducing the carbon footprint of retail products and aligning with environmentally conscious practices.

In recent studies, CDs produced via the top-down thermal decomposition from renewable sources have been incorporated into various composite materials, including plant gum (*V. nilotica*)^97^ embedded in a chitosan/gelatin matrix, *Sophora japonica* extract-based CDs in gelatin,^98^ yeast-derived CDs into nanocellulose membranes,^99^ and pomelo peel-derived CDs in a gelatin/alginate dialdehyde matrix.^100^ These formulations demonstrated effective antifungal properties, primarily through photodynamic activation of the CDs, which generate ROS. This approach offers a cost-effective and nontoxic solution for enhancing the preservation of perishable foods.^101^ Films incorporating CDs synthesized from lemon and onion juice through an eco-friendly hydrothermal process have been reported.^102^ From a structural point of view, the absence of crystallinity in the onion-derived CDs, as opposed to the lemon-based CDs, suggests a higher concentration of oxygen-containing functional groups on the surface, such as −OH and −COOH, groups, as confirmed by FTIR spectroscopy. These green-fluorescent CDs were applied to the surface of fresh strawberries to create active packaging films aimed at extending the shelf life of strawberries by inhibiting fungal growth (Figure 6d). The resulting films demonstrated significant antifungal activity against common strawberry pathogens, particularly *B. cinérea*, effectively reducing mold growth and decay during storage, especially for onion-derived CDs. Additionally, the incorporation of CDs enhanced the mechanical properties and UV-blocking capabilities of the films, contributing to improved preservation of the fruit’s quality. This type of approach not only offers a sustainable method for utilizing natural waste products but also provides a promising solution for active food packaging applications. Using biological transmission electron microscopy (Bio-TEM), the aforementioned HACC-based CDs were able to effectively damage fungal cell structures, inhibiting growth.^94^ This structural damage suggested that the CDs disrupt fungal cell integrity, leading to the death of *C. versicolor*, a well-known decay fungus with strong wood-degrading capabilities. In fact, these hydrothermally synthesized CDs served as an efficient and eco-friendly wood preservative, significantly enhancing wood durability and lifespan against the mentioned fungi. These results provide valuable insights for the future development of nanotechnology-based wood preservatives.

### 4.3 Labeling and Bioimaging Applications

A significant contribution to the field of bioimaging materials for fungal detection emerged from the Kailasa group among others. In 2015, Kailasa’s team reported the hydrothermal synthesis of different multifluorescent and well dispersed CDs (∼3.5 nm) using pomegranate,^103^ papaya,^104^ or apple^105^ juice as natural and renewable precursors (Figure 7a). Interestingly, the authors observed that the optimum reaction time was 12 h to obtain CDs with the highest fluorescence intensity without the need for additional surface passivation. These CDs were effectively used as probes for imaging *F. avenaceum*,^103^ *A. aculeatus*,^104^ or *M. oryzae* ^105^ cells, as evidenced by confocal fluorescence microscopy images showing distinct green and red fluorescence within the fungal cells. The biocompatibility of these CDs with multicolor emission was also highlighted, suggesting the potential of fruit juice-derived CDs as bioimaging agents for various biomedical applications. More recently, Kailasa and co-workers reported the hydrothermal synthesis of CDs using *Manilkara zapota* (sapodilla) fruits utilizing sulfuric and phosphoric acids as oxidizing agents to tune the emission properties of the CDs, resulting in blue, green, and yellow fluorescent nanomaterials (Figure 7b).^106^ Characterization of the synthesized nanoparticles revealed FQY and an average particle size of 5.7% and 1.9 ± 0.3 nm for blue, 7.9% and 2.9 ± 0.7 nm for green, and 5.2% and 4.5 ± 1.25 nm for yellow CDs, respectively. The biocompatibility of these multicolor emissive CDs was assessed through cytotoxicity studies on HeLa cells, confirming their nontoxic nature. Effective bioimaging of fungal (*Aspergillus aculeatus* and *Fomitopsis* sp.) cells with green and yellow CDs, respectively, was demonstrated, with CDs being well-distributed within both the cell membrane and cytoplasm. These CDs emitted fluorescence in the blue, green, and red wavelengths when excited at 405, 488, and 561 nm, respectively, as shown in Figure 7c. Importantly, no fluorescence emission was detected in cells without CDs treatment, indicating that the CDs efficiently penetrate the cells via endocytosis and achieve a high degree of biodistribution.

**Figure 7:**
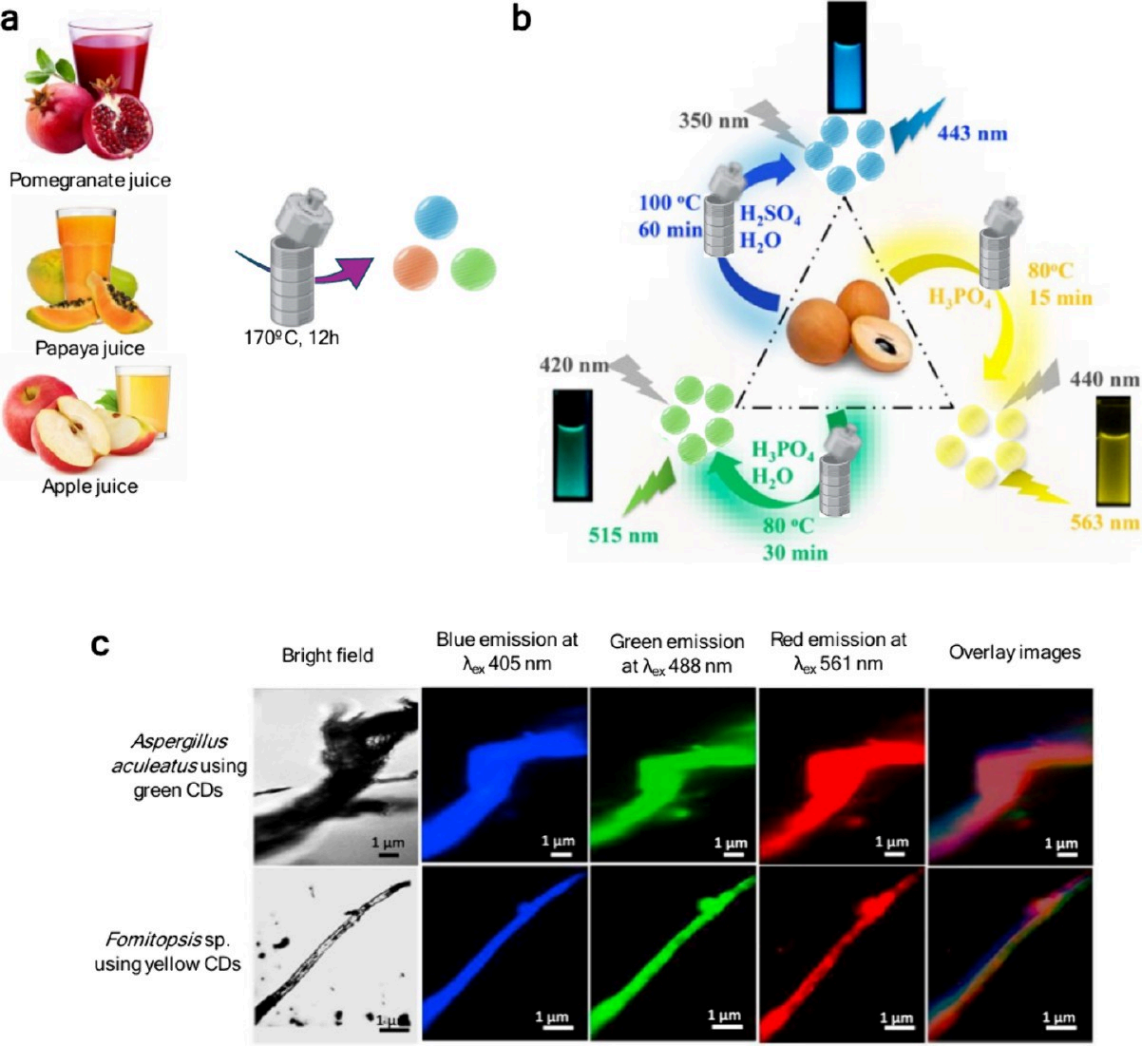
(a) Graphical representation of the CDs’ synthetic approach from pomegranate, papaya, or apple juice. (b) Graphical representation of the CDs’ synthetic approach adapted from Bhamore et al.^106^ (c) Confocal fluorescence microscopic images of *A. aculeatus* and *Fomitopsis* sp., at excitation wavelength of 405, 488, and 561 nm. Image adapted from Bhamore et al.^106^

CDs produced from pea and sesame as a tool for tracking fungal infections in living organisms was reported by Yang and co-workers.^107^ Particularly, CDs equipped with the polar groups −OH, −NH_2_, and −COOH from pea were found to be excellent fluorescent probes for specific binding to *C. neoformans*, rather than other cells, fungi, or bacteria. *In vivo* experiments in which mice’s lungs were infected with *C. neoformans* were undertaken on account of the favorable properties of the nanomaterials, which included strong blue fluorescence, low particle size distribution (3.1–4.3 nm), biocompatibility, and low cytotoxicity. Upon administration of CDs, the positions of the fungus within the mice could be traced through fluorescence imaging (Figure 8a).

**Figure 8:**
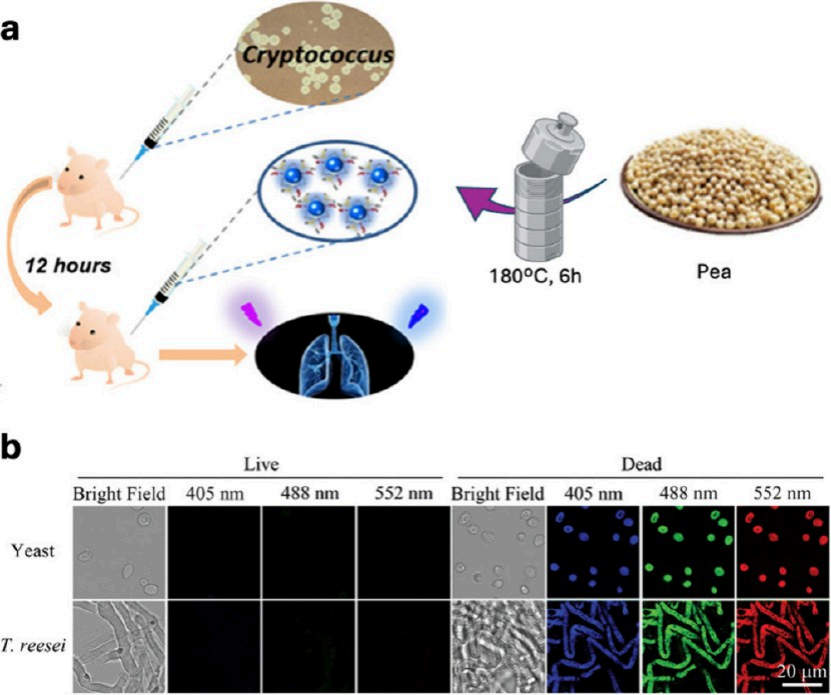
(a) Graphical representation of the CDs’ synthetic approach and labeling of the infected lung by *Cryptococcus neoformans in* vivo. Image adapted from Su et al.^107^ (b) Confocal fluorescence microscopic images of live and dead fungal cells (yeast and *T. reesei*) stained with *S. aureus*-based CDs, at excitation wavelength of 405, 488, and 552 nm. Image reproduced from Hua et al.^108^

As discussed previously, Wu and co-workers reported the synthesis of CDs produced from small and well-defined molecular precursors (i.e., rose bengal and d,l-cysteine)^60^ capable of discriminating between live and dead cells. The same group also reported a green synthesis of CDs produced from bacterial sources (*S. aureus* or *E. coli* cells) capable of differentiating between live and dead fungal cells.^108^ These CDs demonstrated remarkable stability (even after several months of storage at approximately 4 °C) and aqueous dispersion. The robustness of the CDs can be attributed to the highly negative surface charge (associated with the presence of carboxyl groups), which results in strong electrostatic repulsion between the CD nanoparticles. As expected, these CDs possess a highly negative surface charge (zeta potential around −40 mV) and an appropriate size of a few nanometers, enabling them to selectively stain dead fungal cells, including *S. cerevisiae* (yeast) and *T. reesei*, without affecting live cells. After a 1 h incubation with these CDs, only the dead yeast and *T. reesei* exhibited strong blue, green, and red fluorescence when excited at 405, 488, and 552 nm, respectively, whereas the live fungal cells remained unstained by the CDs (Figure 8b). This selective staining facilitates effective differentiation between the live and dead states of the fungal cells.

Many reports of CDs generated using sustainable starting materials which undergo a hydrothermal treatment and that exhibit a dual functionality, as, for example, in metal ion sensing and bioimaging of fungal cells, have been reported in the literature. In 2017, Lee and co-workers described a simple hydrothermal-carbonization method to synthesize CDs utilizing *Chionanthus retusus* fruit extract and aqueous ammonia as carbon and nitrogen sources, respectively.^109^ HRTEM revealed that the CDs had an average size of approximately 5 ± 2 nm, with an interlayer distance of 0.21 nm. The CDs exhibited durable fluorescence properties with a quantum yield of 9%, with rich nitrogen (amine), carbonyl (carboxylic acid), and hydroxyl (OH) groups on the surface. In fact, the rich nitrogen and oxygen on the CD’s surface allowed to detect Fe^3+^ ions with high sensitivity and selectivity. More interestingly, fluorescence microscopy studies using fungal strains (*C. albicans* and *C. neoformans*) indicated that the blue-fluorescent CDs could permeate cell membranes effectively, allowing for differential staining of these prokaryotic and eukaryotic cells with negligible cytotoxicity. This dual functionality highlights their potential application in metal ion sensing, particularly for Fe^3+^, and holds potential in biological applications such as bioimaging. CDs from *Tagetes patula* flowers was also used as a fluorescent probe for detecting this type of ion and as antifungal agent (*M. oryzae*).^110^ Similarly, Bhamore and co-workers synthesized CDs using *Acacia concinna* seeds, commonly known as shikakai, as a green precursor for the selective detection of copper ions (Cu^2+^) and for imaging fungal cells.^111^ Alternative renewable resources, such as plastic waste (plastic bags, plastic cups, and plastic bottles), have been used for the preparation of carboxylic acid/hydroxy-coated CDs for the same purposes.^112^ The Bhamore group’s CDs were synthesized via a rapid microwave heating process, resulting in ultrasmall particles with notable fluorescent properties.^111^ Particularly, these CDs were able to selectively sense the Cu^2+^ ion through strong chelation between the Cu^2+^ ion and both amine and hydroxyl groups of CDs. Beyond metal ion detection, the biocompatibility of the synthesized CDs was assessed through multicolor (blue, green, and red) imaging of *Penicillium* sp. fungal cells. The CDs successfully penetrated the fungal cell walls via endocytosis, emitting bright fluorescence that facilitated clear visualization in the cytoplasm, and especially in the nuclei of cells. More recently, two different multicolour emissive CDs and nitrogen-doped CDs have been prepared from *Panax notoginseng* as a natural carbon source for fungal bioimaging and high selectivity detection of Cr^6+^.^113^ Interestingly, N-rich CDs containing hydrophilic groups such as −OH, −NH_2_, and −COOH on the CD surface exhibited a smaller average particle size and stronger photoluminesce emission than the corresponding CDs without the N-doping agent, as well as higher water solubility and photostability. The authors attributed this difference between these two different nanomaterials to the presence of N atoms codoped on the surface of the CDs.

Similar to the example discussed in Section 3.3 of citric acid-based CDs for detecting the pathogenic fungus *C. albicans* developed by Li et al.,^76^ blue-fluorescent CDs synthesized from cornstalk using a hydrothermal method were modified with water-soluble and well-known antifungal agent AmB on their surfaces to enhance their specificity toward *C. albicans*.^114^ The CDs-AmB conjugates demonstrated a linear detection range for *C. albicans* from 2.60 × 10^5^ to 1.99 × 10^8^ colony-forming units per milliliter (cfu/mL), with a detection limit of 1124 cfu/mL. This performance indicates a high sensitivity suitable for practical applications such as food safety and clinical diagnostics. However, the high cost of water-soluble AmB could limit their large-scale application. To address this, the researchers explored the use of alcohol-soluble AmB in subsequent experiments, indicating its potential to broaden the method’s applicability for fungal detection.

Another successful example of *C. albicans* tracking is represented by the work of Pandey et al.^115^ The authors reported the synthesis of 2–3 nm hydrophilic CDs with blue fluorescence, derived from a salmon DNA precursor, using a hydrothermal method at a 2% (w/v) DNA concentration. The DNA-based CDs were synthesized by using a carbonization method that retains the inherent properties of DNA, allowing for stable and tunable fluorescence. These CDs exhibited highly efficient internalization *C. albicans* with minimal cytotoxicity, excellent photoluminescence stability, and high biocompatibility, making them promising nanotrackers for microbial studies.

A microwave-assisted pyrolysis method from cost-effective and sustainable carbon sources was also used to rapidly produce highly luminescent CDs for fungal bioimaging of *C. albicans* ^116^ and other fungus.^117^ For instance, starting from tender coconut water, CDs measuring between 1–6 nm in size (depending of the reaction temperature), that exhibited blue and green fluorescence when excited at 390 and 450 nm wavelengths, were obtained within a minute of MW treatment.^118^ These CDs were successfully utilized for bioimaging of fungal cells, namely, *A. niger*. Fresh tomato pulp was also used to prepare blue-fluorescent CDs with tunable optical properties. Nucleophiles such as ethylenediamine (EDA) and urea were introduced during the synthesis to modulate the chemical composition and enhance the fluorescence properties of the resulting CDs.^119^ Characterization of the CDs revealed that those synthesized with urea exhibited a highly crystalline structure, minimal amorphous surface content, and particle sizes smaller than 5 nm. The incorporation of nitrogen from urea contributed to the formation of a cyclic core with strong electron-withdrawing capabilities within the conjugated carbon plane. This structural configuration led to pronounced quantum confinement effects, resulting in UV fluorescence emission. In contrast, CDs synthesized using only fresh tomato or with the addition of EDA produced larger particles (>20 nm) and exhibited fluorescence primarily governed by surface states. Practical applications of these CDs stemming from fresh tomato pulp in the presence of EDA and urea were also explored, demonstrating their efficacy in bioimaging plant pathogenic fungi such as *C. gloeosporioides*, *V. mali*, and *B. berengeriana*.

## 5 Metallic and Nonmetallic Heteroatom-Doped CDs and Composites and Applications

### 5.1 Antifungal Applications

One of the key determining factors contributing to the biocompatibility of CDs is their composition, which primarily consists of carbon, hydrogen, and oxygen. As mentioned in the introduction, the physicochemical, optical, and biological properties of CDs can be precisely modulated by the selection of the appropriate precursors, the choice of synthetic method, or postsynthesis modifications. This extends to the incorporation of heteroatoms, whether metallic or nonmetallic (Table 3). For example, metal doping can introduce paramagnetic behavior, unique light absorption properties, or enhance heating capabilities, making them suitable for specific biomedical applications. Additionally, doping plays a vital role in addressing microbial infections, as the interaction between nanomaterials and pathogens is strongly influenced by factors such as nanoparticle surface polarization, chemical composition, functional groups, and amphiphilic properties.^32^

**Table 3:** Metallic and Nonmetallic Heteroatom-Doped CDs and Composites and Their Applications

carbon source	doping agent or composite	synthetic method	fungal species	applications	ref.
carboxymethyl cellulose	AuCl_3_ or PtCl_4_	hydrothermal (infrared irradiation)	*C. albicans*	antifungal	^120^
citric acid and PEG	HAuCl_4_	hydrothermal	*C. albicans*	antifungal	^121^
EDA, 3-iodo-tyrosine and CuCl_2_	-	hydrothermal	*C. albicans*	antifungal	^122^
polyethylenimine and citric acid	AgNO_3_	hydrothermal	*S. cerevisiae*	antifungal	^123^
orange peel	silver nanoparticles (AgNPs) from AgNO_3_	hydrothermal	*A. niger*	antifungal	^124^
onion	AgNPs from AgNO_3_	hydrothermal	*Rhizopus* sp.	antifungal	^125^
			*Penicillium* sp.		
			*C. albicans*		
			*Aspergillus* sp.		
citric acid and *o*-phenylene-diamine	nickel oxide nanoparticles (NiO NPs)	hydrothermal	*C. albicans*	antifungal	^126^
propanol	Ti^3+^, TiO_2_, and Pd nanocomposites	hydrothermal	*F. graminearum*	antifungal	^127^
			*C. gloeosporioides*		
			*B. dothidea*		
			*F. moniliforme*		
			*F. oxysporum*		
alanine and citric acid	hematite (α-Fe_2_O_3_), hydroxypropyl cellulose cross-linked chitosan (HPCCS) and ulvan (UN) nanoparticles	microwave	*A. niger*	antifungal	^128,129^
			*C. albicans*		
polyethylenimine and citric acid	silica nanoparticles and dialdehyde chitosan (DCS)	hydrothermal	-	antimildew	^128,129^
citric acid	urea	solvothermal (DMF)	*C. albicans*	antifungal, fabric functionalization	^130^
chitosan	chitosan/pectin	hydrothermal	*Colletotrichum* sp.	antifungal, food packaging	^131^
glucose	urea	solvothermal	*A. flavus*	antifungal, food packaging	^132^
glucose	-	hydrothermal	*A. flavus*	antifungal, food packaging	^133^
soy-protein isolate	AgNPs composite	hydrothermal	*R. stolonifera*	antifungal, food packaging	^136^
banana juice	Cu_2_O composite	hydrothemal	*C. albicans*		^137^
polyethylene glycol or lemon salt or paraphenylenediamine	urea	hydrothemal or solvothermal (DMF)	*C. albicans*	antifungal, food packaging	^138^
glucose	-	hydrothermal	*A. flavus*	antifungal, food packaging	^139^
			*C. orbiculare*		
glucose	urea	hydrothermal	*A. fumigatus*	antifungal, food packaging	^140^
			*A. flavus*		
			*F. solani*		
			*P. citrinum*		
			*C. albicans*		
			*R. rubra*		
ascorbic acid	chitosan	hydrothermal	*A. niger*, *P. chrysogenum*	antifungal, food packaging	^141^
dried mustard powder MOF composite	-	hydrothermal	*A. flavus*, *P. chrysogenum*	antifungal, food packaging	^142^
eggplant peel powder	-	hydrothermal	*A. flavus*, *P. chrysogenum*	antifungal, food packaging	^143^
EDTA, l-cysteine and Eu(NO_3_)_3_	-	microwave	*Fomitopsis* sp.	bioimaging	^144^
histidine	3-aminopropyl-triethoxysilane and Eu(NO_3_)_3_	hydrothermal	*C. albicans*, *C. parapsilosis*, *C. tropicalis*	bioimaging, antifungal, anticounterfeiting	^145^
citric acid	-	combustion process liquid–liquid interface	spores of *A. niger*, *P. chrysogen*	bioimaging	^146^

For instance, antimicrobial activity, among other biological applications, of Au- and Pt-based CDs was studied and compared to carboxymethyl cellulose-based undoped CDs.^120^ CDs obtained from carboxymethyl cellulose were modified with either AuCl_3_ (Au-based CDs) or PtCl_4_ (Pt-based CDs) by hydrothermal treatment using infrared irradiation. Transmission electron microscopy revealed that the base CDs had an average size of 8.7 nm. Au modification resulted in a negligible size increase to 8.9 nm, while Pt doping led to a more noticeable enlargement to 12.4 nm. After metal modification, the zeta potential decreased to −2.8 mV for Au-based CDs and −5.3 mV for Pt-based CDs compared to −21.9 mV for undoped CDs, suggesting reduced stability due to weak van der Waals forces. It was also found that Au modification resulted in a greater decline in stability compared to Pt doping. Therefore, Au doping appears to be more suitable for nucleating highly stable metal-modified CDs. Biological assessments indicated that Pt-based CDs exhibited superior anti-inflammatory effects, maintaining a cell viability of 78%, and enhanced antimicrobial properties with a 91% reduction of *C. albicans* compared to 70% and 89.8% for undoped CDs and Au-based CDs, respectively. Conversely, Au-based CDs showed a higher anticancer potential, reducing the cell viability by 83%. These findings suggest that the choice of metal dopant in CDs can be strategically utilized to tailor their interactions with the environment and biomedical applications, with Au enhancing anticancer activity and Pt improving anti-inflammatory and antimicrobial effects of carboxymethyl cellulose-based CDs. Moreover, superior antifungal activity was found overall for metal-doped CDs compared to that of CDs. Alternatively, Au-based CDs from citric acid and PEG as precursors and HAuCl_4_ as gold dopant were also used to explore the size-dependent effects on toxicity of these CDs against *C. albicans*.^121^ The authors determined that smaller-sized Au-based CDs had significant antifungal activity against *C. albicans*, whereas larger nanomaterials were less effective. The MIC_80_ values for the smaller Au-based CDs with diameters ranging from 22 ± 2 to 30 ± 2 nm were 250–500 μg mL^–1^, while larger Au-based CDs with diameters greater than 30 nm did not exhibit any toxicity.

Another example of metal-doped CDs with potential as antifungal agents against *C. albicans* is represented by the work from Yang and co-workers.^122^ Copper- and iodine-doped carbon dots (Cu/I-based CDs) were synthesized from 3-iodo-tyrosine, CuCl_2_, and ethylenediamine via a one-step hydrothermal method (Figure 9). These Cu/I-based CDs exhibited intrinsic peroxidase-like activity, enhancing the decomposition of hydrogen peroxide (H_2_O_2_) to generate hydroxyl radicals (^•^OH), effectively killing *C. albicans*. At an exogenous H_2_O_2_ concentration of 0.5 mM, a Cu/I-based CD concentration of 0.585 mg/mL achieved a 10% survival rate of *C. albicans* after 90 min of LED irradiation (16 W). The Cu/I-CDs, which mimic nanozymes, effectively destroyed *C. albicans* biofilms without causing significant toxicity *in vitro* and *in vivo*. In mouse models, these nanozymes accelerated the death of *C. albicans*, promoting wound healing and treating vulvovaginal candidiasis. High biocompatibility was also confirmed through hemolysis and MTT toxicity assays.

**Figure 9:**
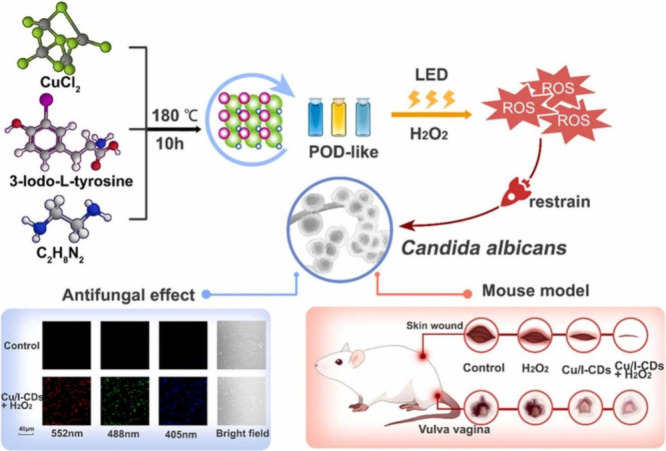
Synthesis of Cu/I-based CDs and their antifungal action against *C. albicans* infection. Image reproduced from Li et al.^122^

The choice of the synthetic route also has an effect on both physicochemical properties of the CDs and their antimicrobial activity. Zhao and co-workers synthesized two types of blue light-emitting Ag-doped CDs using polyethylenimine, citric acid, and silver nitrate (AgNO_3_) as raw materials.^123^ One of them was produced via a one-step synthesis (Ag-doped CDs-1), while the second one was obtained through a two-step process (Ag-doped CDs-2) (Figure 10). The authors suggested two possible mechanisms for the synthesis of these two types of CDs. For the Ag-doped CDs-1 strategy, Ag was doped onto the surface and interior of the CDs in the form of Ag^+^, whereas in the case of Ag-doped CDs-2, the polyethylenimine-based CDs are formed first, while Ag^+^ is then subsequently reduced to Ag NPs in the presence of formaldehyde and attached onto the surface of the carbon core to form the core–shell structure of Ag-doped CDs-2. Characterization revealed differences between Ag-doped CDs-1 and CDs-2 in terms of emission wavelength, surface groups (Ag-doped CDs-2 are amine-richer), particle size, quantum yields (25.4% and 2.1% for Ag-doped CDs-1 and CDs-2, respectively), surface charges (Ag-doped CDs-1 is positively charged, whereas Ag-doped CDs-2 is more negative), and elemental content. Notably, Ag-doped CDs-2 exhibited a longer emission wavelength, larger particle size, higher Ag content, and greater antimicrobial (both antibacterial and antifungal) efficacy compared with Ag-doped CDs-1.

**Figure 10:**
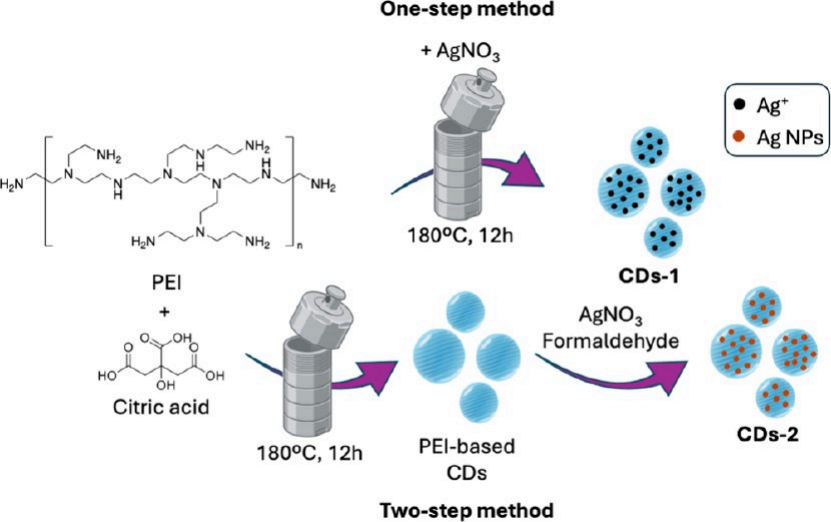
Synthesis of Ag-based CDs using two different synthetic strategies. Image adapted from Zhao et al.^123^

In addition to metal-doped CDs with intrinsic antifungal properties, various metallic CD-based nanocomposites, i.e., CDs embedded in a metal or polymer nanomaterial, have been designed as antifungal agents. The rich presence of surface functional groups and π-domains within their structure enables CD functionalization. Postsynthetic modifications of CDs are typically achieved through physical adsorption via hydrophobic/hydrophilic interactions or hydrogen bonding, as well as through chemical functionalization.

For instance, nanocomposites based on CDs and silver nanoparticles (AgNPs) by a hydrothermal reaction have been reported for antifungal applications. Tenkayala and colleagues developed AgNPs@CDs composites by utilizing the reducing properties of CDs, derived from orange peel, to facilitate the formation of AgNPs from AgNO_3_ through solution heating using a sand bath (Figure 11a).^124^ The interaction between Ag^+^ ions and hydroxy (−OH) groups, along with the reduction contribution of oxygen-rich functional groups, enabled the simultaneous generation and stabilization of AgNPs *in situ* without the need for external reducing agents. The antifungal activity of the AgNPs@CDs was assessed using the agar diffusion method against *A. niger*. The strongest inhibition zone (40 mm) was observed when the strain was treated with 100 μL of a 1 mg/mL AgNPs@CDs solution, highlighting their potential application in antifungal treatments. In a different study, Slewa et al.^125^ synthesized AgNPs@CDs composites using onion-derived CDs (discussed in Section 4.2)^102^ and AgNPs from AgNO_3_ through solution heating using an autoclave method. Similar to orange-based CDs, onion-derived CDs function as both reducing and stabilizing agents in the formation of AgNPs@CDs nanocomposites. By varying CDs’ concentrations during the hydrothermal treatment, the authors controlled the size and morphology of AgNPs. It was found that with higher CDs concentrations, smaller nanoparticles (from 37.03 to 7.8 nm) were obtained. The antimicrobial efficacy of AgNPs@CDs was assessed using the agar-well diffusion method against fungal pathogens, including *Rhizopus* sp., *Penicillium* sp., *C. albicans*, and *Aspergillus* sp. As previously observed for Au-based CDs,^121^ these nanocomposites demonstrated stronger antifungal activity as their size decreased, with inhibition zones for S4 (the smallest AgNPs@CDs) of 36.03 ± 3.1, 24.99 ± 1.4, 39.67 ± 2.8, and 35.08 ± 2.1 mm against *Rhizopus* sp., *Penicillium* sp., *C. albicans*, and *Aspergillus* sp., respectively (Figure 11b).^125^

**Figure 11:**
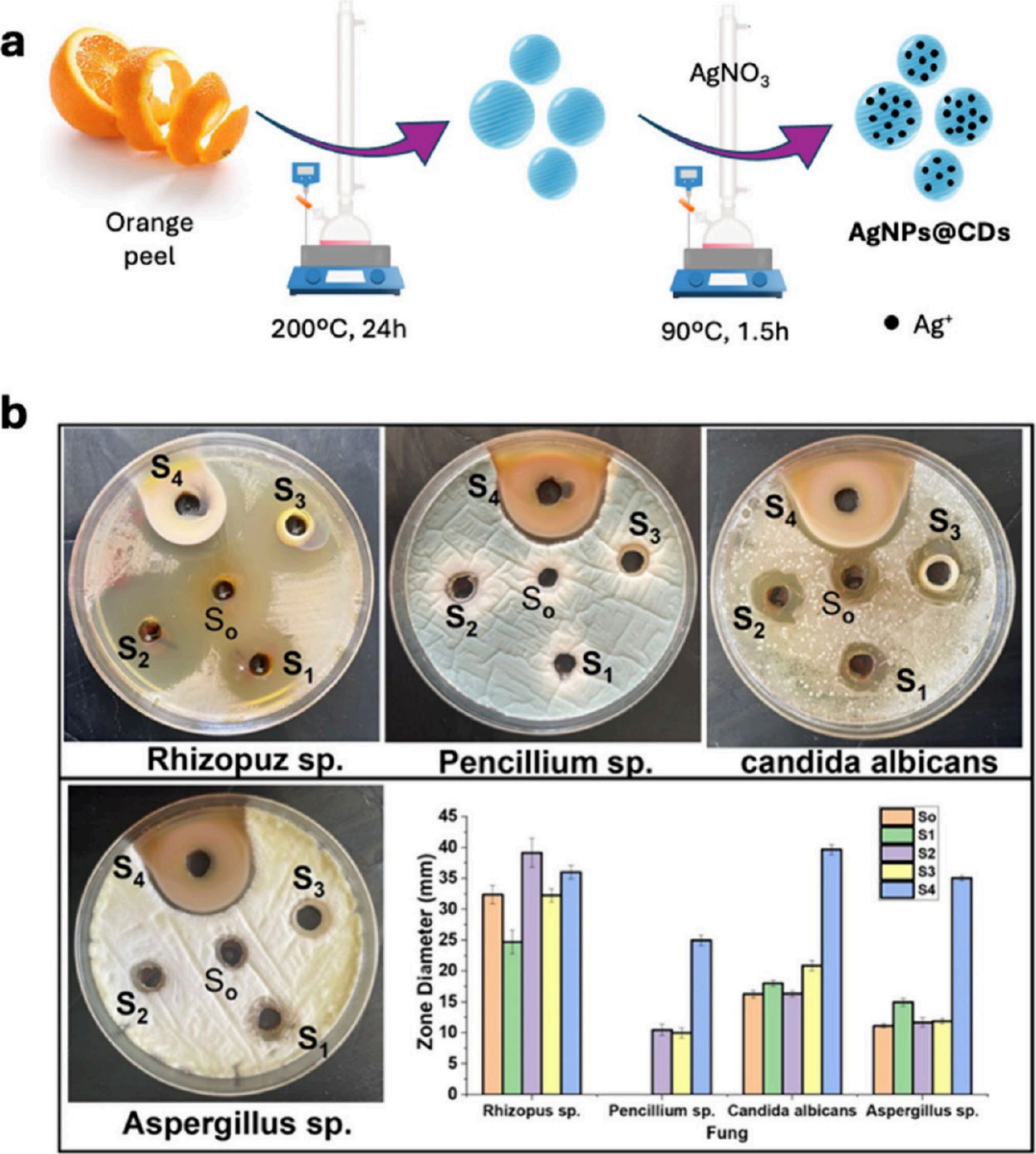
(a) Synthesis of AgNPs@CDs composites from CDs derived from orange peel. (b) Zones of inhibition of the antifungal activity of AgNPs@CDs composites and colloidal solutions of AgNPs in CDs from CDs derived from onion juice against different fungi with a bar graph. Image reproduced with permission from Slewa et al.^125^

The incorporation of blue emitting CDs into metal nanoparticles also enhanced their antifungal activity compared to the metal oxide nanoparticles counterparts. Two representative examples come from the work of the Dejene and Shi teams. In 2023, Etefa et al.^126^ reported the green synthesis of a biosynthetic composite CDs@NiO NPs using nickel oxide nanoparticles (NiO NPs) and CDs for antimicrobial applications. NiO NPs were synthesized using *Croton macrostachyus* leaf extract and nickel nitrate, while CDs were produced from citric acid and *o*-phenylenediamine. The average particle sizes of NiONPs and CDs@NiO NPs were 25.34 ± 0.12 and 24.95 ± 0.22 nm, respectively. However, the incorporation of CDs increased the composite’s surface roughness and surface area, enhancing its antimicrobial properties. In fact, the composite CDs@NiO NPs demonstrated strong inhibitory effects against *C. albicans*, with inhibition zones of 24 mm for CDs@NiO NPs, outperforming NiO NPs alone (22 mm). On the other hand, Shi’s work presented an innovative approach to enhancing the antimicrobial properties of titanium dioxide (TiO_2_) through Ti^3+^ self-doping and comodification with CDs and Pd nanocomposites.^127^ By addressing the limitations of conventional TiO_2_, such as limited visible-light absorption and rapid charge recombination, the authors aimed to develop a highly efficient disinfection material with improved photocatalytic activity with efficient photodisinfection of five pathogenic agricultural fungi. The Ti^3+^ self-doping process significantly improved the material’s light absorption capacity and charge separation efficiency. The incorporation of CDs further facilitated electron transfer, while the addition of Pd nanoparticles to the titanium/CDs composite, functioned as cocatalysts, enhancing the catalytic performance of the material. These modifications resulted in superior ROS generation, which plays a crucial role in microbial disinfection. The study demonstrated that the Ti^3+^/CDs/Pd@TiO_2_ nanocomposite exhibited remarkable antibacterial and antifungal activity against various pathogens, outperforming traditional TiO_2_-based materials.

More complex doped CD-based nanocomposites with superior antifungal properties have also been designed.^128,129^ For instance, nanocomposites generated by combining hematite (α-Fe_2_O_3_) and CDs from alanine and citric acid through microwave-assisted method, have been reported.^128^ These metal-doped CDs, measuring 3–5 nm, were encapsulated within hydroxypropyl cellulose cross-linked chitosan (HPCCS) and ulvan (UN) nanoparticles. The resulting nanocomposites (α-Fe_2_O_3_@CDs)@HPCCS/UN exhibited unique pH-responsiveness and optical properties, including single-excitation (440 nm) and dual-emission fluorescence (505 and 628 nm for green and red light from α-Fe_2_O_3_@CDs and HPCCS/UN, respectively). The team demonstrated efficient and pH-responsive drug delivery, releasing ulvan rapidly at pH 7.4 and more slowly in acidic conditions. The nanocomposites (α-Fe_2_O_3_@CDs)@HPCCS/UN exhibited stronger antifungal efficiency against *A. niger* and *C. albicans* than α-Fe_2_O_3_@CDs. In fact, the antifungal activities of (α-Fe_2_O_3_@CDs)@HPCCS/UN and α-Fe_2_O_3_@CDs were measured as ∼18 and 10 mm for *A. niger* and ∼22 and 12 mm for *C. albicans*, respectively, at a concentration of 50 μg per well. A similar trend was observed against bacterial strains.

Inspired by oysters, a multifunctional organic–inorganic hybrid soybean meal (SM)-based nanocomposite was developed by incorporating amino-modified CDs functionalized silica nanoparticles (CDs@SiO_2_) and dialdehyde chitosan (DCS) into the SM matrix (Figure 12a).^129^ The synthesized CDs@SiO_2_ nanocomposite, containing amino groups, enhanced the dispersion of SiO_2_ within the organic SM matrix by providing additional active sites. Acting as a glue molecule, DCS promoted strong interfacial interactions between CDs@SiO_2_ and the SM matrix, forming a stable connection through the Schiff base reaction. The biomimetic organic–inorganic structure was designed to impart superior performance to the soy protein adhesive, benefiting from its rigid framework and strong cohesion effects, including electrostatic interactions, hydrogen bonding, reversible imine bonds, and nonreversible covalent bonds. The nanocomposite MSM/DCS/CDs@SiO_2_ demonstrated excellent antimildew and antibacterial properties due to the incorporation of DCS.

**Figure 12:**
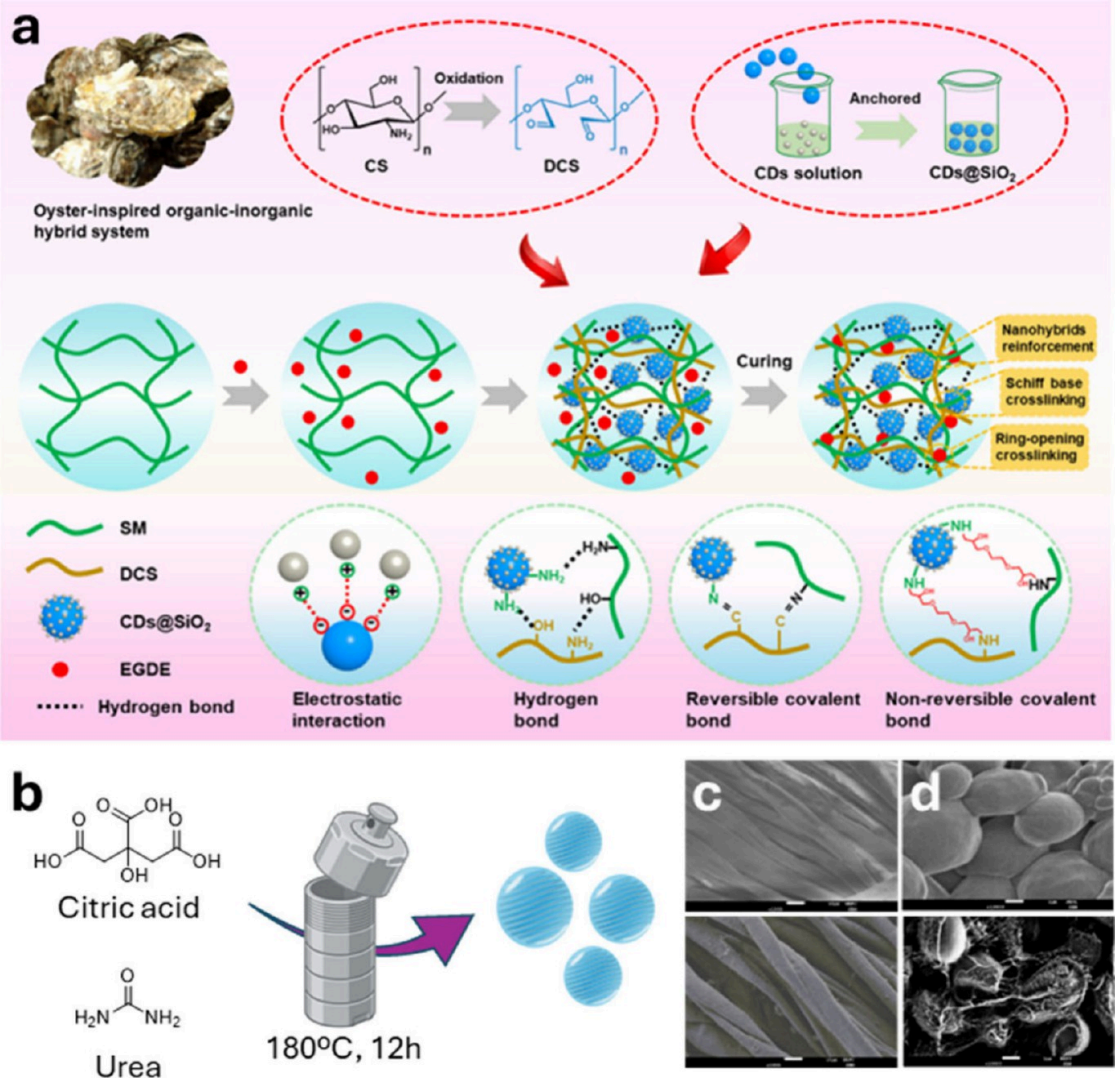
(a) Oyster-inspired design to fabricate the MSM/DCS/CDs@SiO_2_ adhesive and reaction mechanism. (b) Graphical representation of the CDs’ synthetic approach adapted from Evseev et al.^130^ (c) SEM image at 1000x magnification of pristine cotton fabric (top) and CD-coated cotton fabric (bottom). (d) SEM image of test cultures of *C. albicans* dried on a SiO_2_ surface after 24 h of incubation. Control (top), and CD treated (bottom). Images (a)–(d) have been produced with permission from Chen et al.^129^

The inclusion of CDs in fabrics has led to the creation of functional materials with good antimicrobial activity. Evseev et al.^130^ synthesized CDs from citric acid and urea using a solvothermal method (Figure 12b) and applied them in textile functionalization (Figure 12c). The CDs exhibited an average size of 8.4 nm measured through AFM and possessed a negative zeta potential of −41.7 mV because of a surface rich in oxygenated species such as carboxyl groups, as demonstrated by FTIR analysis. The composite material made from graphene oxide and CDs, or mild graphene oxide and CDs, exhibited superior antifungal activity when compared to that of the graphene derivatives alone. These materials were chosen because of their known antimicrobial properties (Figure 12d). Cotton fabric was treated by dipping it into an aqueous CD solution, followed by sonication to boost the CD adsorption. Although, CD-functionalized textiles showed a significant loss of CDs after several washing cycles, the combined use of CDs with graphene oxide and mildly oxidized multigraphene resulted in a synergistic effect, which improved antimicrobial activity and increased nanomaterial retention after multiple washes. The antimicrobial properties of this mixture were attributed to the capability of the graphene oxide and mildly oxidized graphene oxide to prevent the agglomeration of CDs upon drying, thus providing a matrix for CDs that was shown to exhibit a higher degree of oxidative stress because of a higher surface morphology.

### 5.2 Biofilm Inhibition and Packaging Applications

As we have already established, CDs have emerged as innovative nanomaterials in nanotechnology, demonstrating significant antifungal properties primarily attributed to their ability to disrupt fungal cell membranes and their susceptibility to photodynamic effects, which promote ROS generation. Their found applications in fungal detection and eradication have highlighted the versatility and potential of these nanoparticles as potent agents against fungal infections with the potential to overcome traditional microbial drug resistance, including examples where CDs have also been incorporated into various composites and matrices to create advanced antifungal functional materials. Beyond antifungal applications, nanocomposite films incorporating CDs for active food packaging^131–133^ or wood preservation^134^ applications have also been explored. In food packaging applications, UV protection is a critical factor to preserve food quality, as exposure to UV radiation can lead to photocatalytic oxidation. This process causes the breakdown and oxidation of nutrients, resulting in off-flavours, rancidity, discoloration, and the formation of toxic derivatives.^135^

For instance, Safitri et al.^131^ developed nanocomposite films using a chitosan/pectin mixture combined with chitosan-based CDs for mango packaging applications. CDs obtained from hydrothermal carbonization of chitosan featured −OH, −NH_2_, and −COOH groups on the surface that allowed the interaction between chitosan/pectin and CDs via hydrogen bonds. These interactions induce aggregation which can lead to a loss of luminescence compared to the blue-emitted CDs alone. The antimicrobial efficacy of these CDs-infused biofilms against *colletotrichum* sp. was assessed using the agar diffusion test, revealing an increase in the inhibition zone with higher CD concentrations (15.5 mm for 1% loading and 24.6 mm for 2% loading). To evaluate their protective function, the chitosan/pectin@CDs were applied to the mango surface and monitored for 24 days. The CDs-biopolymer coatings effectively preserved the fruits by minimizing weight loss and preventing extensive fungal contamination throughout the experiment (Figure 13).

**Figure 13:**
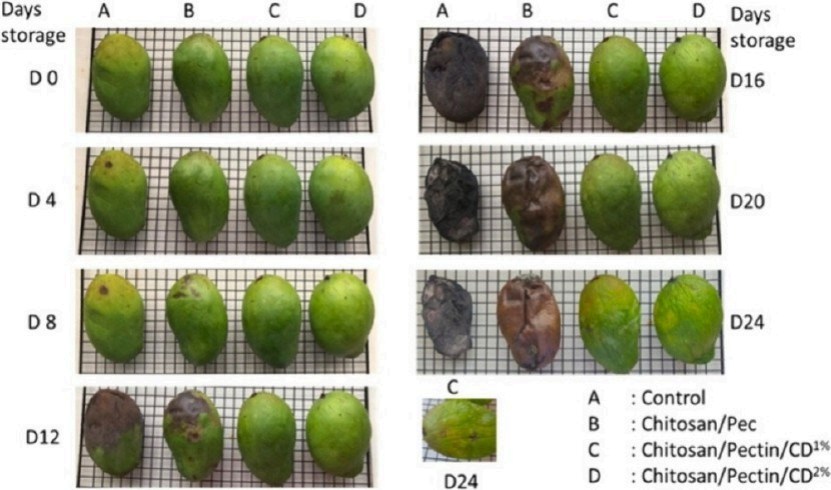
Change in appearance during storage at 25 °C of mangoes coated with chitosan/pectin-based coatings. Image reproduced with permission from Safitri et al.^131^

Pectin matrices were also used for the formation of composite films by integrating glucose-based CDs generated using a hydrothermal approach.^133^ CD-infused pectin films exhibited strong antimicrobial activity, achieving complete eradication of *A. flavus*, and demonstrated a 95% increase in antioxidant activity. The incorporation of the stable and strong luminescent CDs significantly enhanced the film’s UV blocking capabilities, effectively converting UV light into blue light, thereby improving its protective properties. These attributes highlight the promising potential of pectin@CDs for use as transparent fluorescent films in active packaging applications.

Another noteworthy example of protective packaging is the work of Ezati et al.,^132^ who developed thin films based on cellulose nanofibers blended with two types of CDs for potential antimicrobial applications in food packaging. The CDs were synthesized via a one-step hydrothermal process using glucose or a combination of glucose and urea and incorporated into cellulose matrices at a 1 wt % ratio. The antimicrobial performance of these films was tested against *A. flavus*, revealing that the glucose/urea-based CD-cellulose film completely inhibited fungal growth within 2 days, whereas the glucose-based CD-cellulose film exhibited more moderate activity. This highlights the crucial role of doping in enhancing the antifungal properties of the CDs. SEM imaging confirmed that both CD-integrated films severely damaged the fungal membrane, compromising its structural integrity. Furthermore, the authors emphasized that the nitrogen-containing groups in glucose/urea-based CDs enhance the electron-donating capability of the film, leading to the generation of reactive oxygen species that contribute to microbial eradication.

Other CD-based composites have also been developed for food packaging applications. Two notable studies, reported by Koshy et al.^136^ and De et al.,^137^ utilized CDs as metal reductive agents and codopants in the fabrication of Ag- and Cu-functionalized films, respectively. CDs were synthesized from renewable sources through hydrothermal decomposition in Teflon-coated autoclave reactors from soy protein isolate^136^ and banana juice,^137^ respectively, which served as the carbon precursors. In these studies, CDs acted as reducing agents to produce Ag or Cu composites, which were subsequently embedded in chitin nanowhiskers or hyperbranched epoxy matrices. The Cu-CD-epoxy film exhibited remarkable antibacterial and antifungal activity, demonstrating high efficacy against *C. albicans* in disk diffusion assays.^136,137^ Similarly, the Ag-CD-chitin nanowhisker films proved to be effective in inhibiting fungal growth when used for bread packaging, significantly extending shelf life. Additionally, the Ag-CD-chitin films demonstrated reduced water vapor permeability, which underscores their potential as superior coating materials for preserving food texture and organoleptic properties.^136^

In another example, Alaş et al.^138^ synthesized multicolour-emitting CD-poly­(vinyl alcohol) (PVA) composite films using various carbon sources (Figure 14d). Blue-emitting CDs were produced from carob molasses, poly­(ethylene glycol), and urea, while green-fluorescent CDs were synthesized using lemon salt and urea in an H_2_O/ethanol solution. Interestingly, when employing the same carbon sources but using instead DMF as the reaction solvent, yellow-fluorescent CDs were produced. Moreover, red-emitting CDs were prepared starting from paraphenylenediamine. All CDs were synthesized via solvothermal decomposition methods and subsequently integrated into a PVA matrix to create CD-functionalized films (Figures 14e–h). These composite films demonstrated significant antifungal activity by inhibiting the growth of *C. albicans*. To demonstrate their utility of the materials, strawberries dip-coated with the CD-based film showed improved shelf life by minimizing fungal growth, spoilage, and moisture loss, while the films also provided excellent UV-shielding properties (Figure 14i).

**Figure 14:**
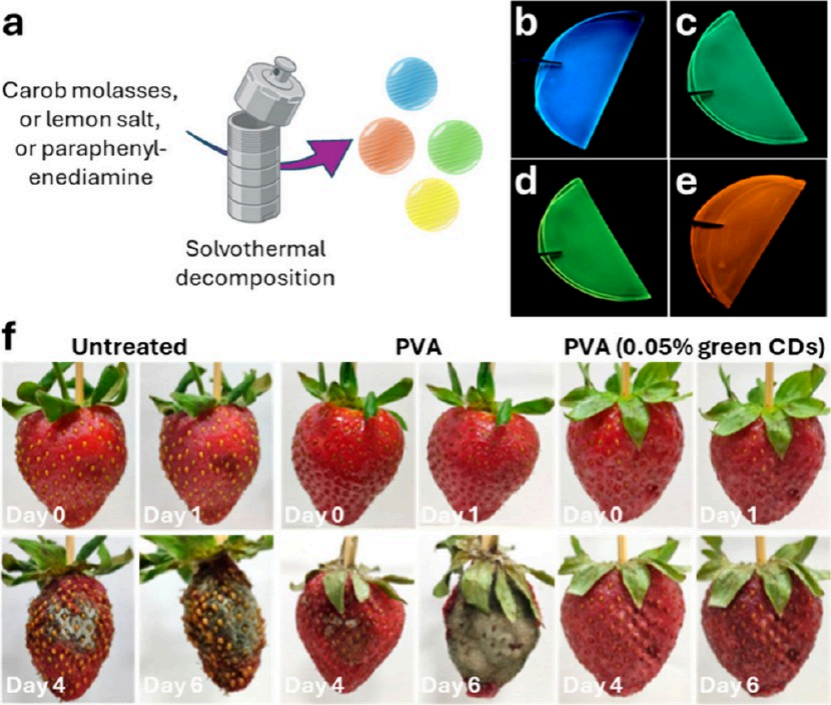
(a) Grapical representation of the CDs’ synthetic approach adopted by Alaş et al.^138^ for the synthesis of different types of fluorescent CDs. (b) Image of blue CD/PVA film under UV light. (c) Image of green CD/PVA film under UV light. (d) Image of yellow CD/PVA film under UV light. (e) Image of red CD/PVA film under UV light. (f) Image of the appearance of untreated, PVA-coated, and CD/PVA coated strawberries at room temperature at varying storage times.

A significant contribution to the field of antifungal materials for fruit preservation emerged from the collaborative efforts of the Rhim and Kim groups. Their studies demonstrated the versatility of CDs synthesized from various carbon sources as potent antifungal agents. The researchers reported the hydrothermal synthesis of glucose-based CDs, using glucose alone^139^ or glucose combined with urea as a the nitrogen dopant source.^140^ In parallel, a range of precursors was explored, including refined molecules such as ascorbic acid^141^ and green sources like dried mustard powder^142^ and eggplant peel,^143^ underscoring the valorisation of food waste materials in these type of systems (Figure 15). The hydrothermal method employed in these examples entails the dispersion of the source materials in water followed by heating in a Teflon-lined reactor using variable temperature and reaction times tailored to each precursor. Following synthesis and purification, CDs were incorporated into various film matrices to produce CD composites. These included chitosan/gelatin, cellulose nanofibers, carboxymethylcellulose (used alone or combined with fish gelatin), and films produced combining CDs with iron-based metal–organic frameworks composites in an agar/gelatin matrix (Figure 15a).^142^ These composite films exhibited excellent antifungal activity and offered innovative and sustainable solutions for enhancing the shelf life and quality of fruit products. As a general outcome, the incorporation of CDs into the matrices did not compromise the optical properties of the films, maintaining transparency within the visible region, which is an ideal feature for food packaging. CD-composite materials have demonstrated excellent UV shielding capabilities, with near-complete UV absorption observed in some cases (Figure 15d).^142^ This property is attributed to the ability of CDs to absorb UV radiation, with a direct correlation between the amount of CD loading in the film matrix and the level of UV shielding achieved.^141^ Additionally, these materials exhibited high antioxidant activity, providing further protection against food degradation. Notably, CD-functionalized materials often displayed improved tensile strength, with enhancements of up to 27% reported in CD-carboxymethyl cellulose-based films.^141^ From a biological perspective, these films exhibited negligible toxicity and strong antimicrobial activity against a range of fungal species, including *A. niger*, *P. chrysogenum*, *A. flavus*, and *C. orbiculare* (Figures 15b–c). Furthermore, when tested as packaging solutions for perishable foods such as tomatoes, grapes, lemons, tangerines, strawberries, and avocados, the results highlight that CD-functionalized materials consistently outperformed both unpackaged and conventionally packaged foods in terms of shelf life (Figures 15e–f).

**Figure 15:**
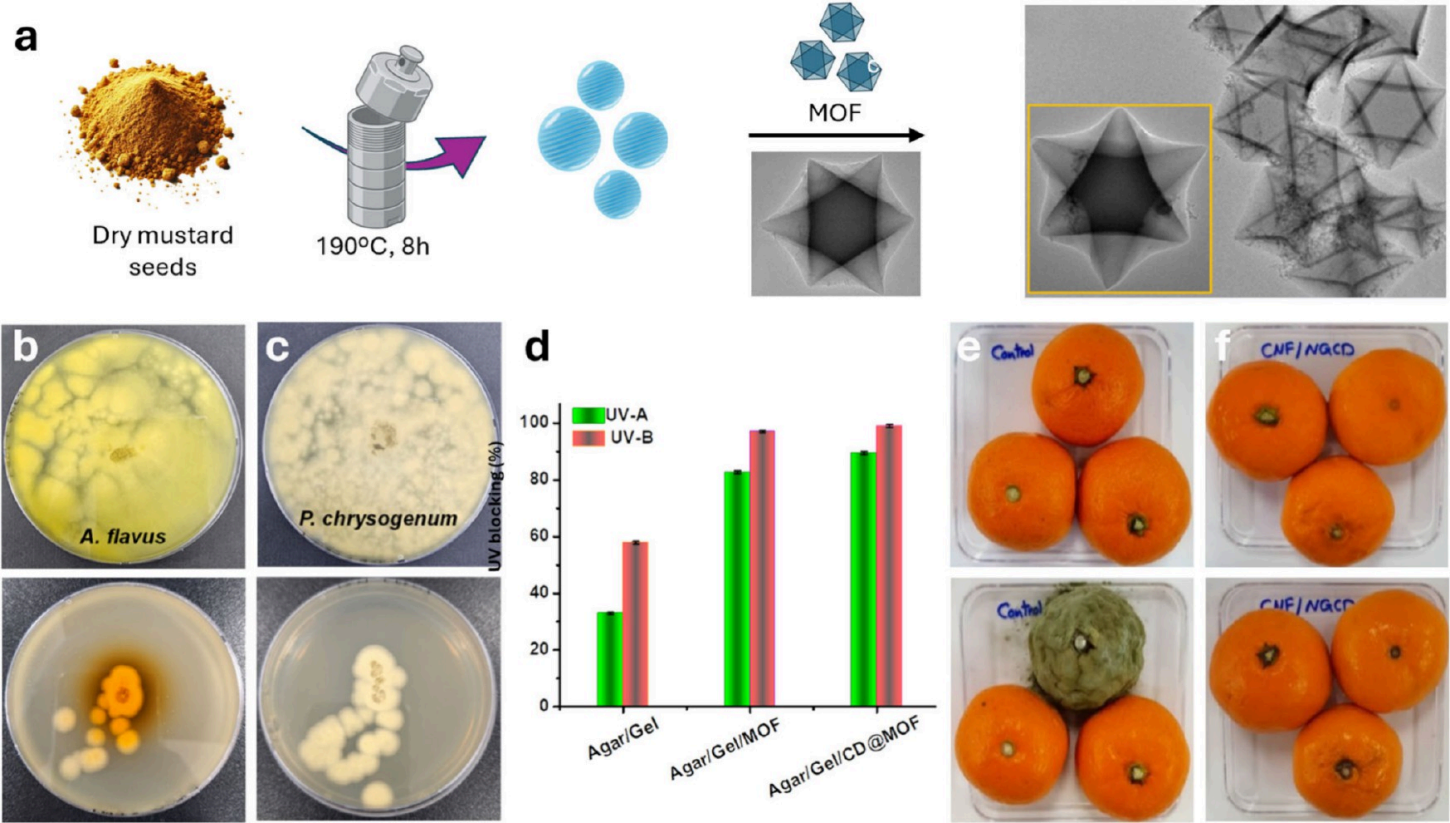
(a) Graphical representation of the CDs’ synthetic approach adopted by Riahi et al.^142^ and Fe-MOF functionalization to achieve CD-MOF composites. (b) Antifungal activity of CD-MOF composites against *A. flavus*.^142^ (c) Antifungal activity of CD-MOF composites against *P. chrysogenum*.^142^ (d) UV-blocking properties of the agar/gel-based films.^142^ (e) Mold growth inhibition assay of uncoated tangerines at day 0 (top) and day 15 (bottom).^140^ (f) Mold growth inhibition assay of tangerines coated with cellulose nanofiber composite functionalized with glucose-based CDs through a dipping process at day 0 (top) and day 15 (bottom).^140^

### 5.3 Labeling and Bioimaging Applications

Kailasa and co-workers synthesized dual functional heteroatom-doped CDs as efficient fluorescence-based sensors for Hg^2+^ ions and effective bioimaging agents for fungal cells.^144^ The researchers employed a rapid and energy-efficient microwave-assisted strategy to prepare the Eu^3+^ hybrid CDs using EDTA, l-cysteine, and Eu­(NO_3_)_3_ as reagents. This method ensures the formation of highly water-soluble, stable, and intensely fluorescent CDs. These CDs exhibited −SH, −OH, −COOH, and −NH_2_ groups on the surface, with a graphite-like nature (lattice fringes with a *d*-spacing value of 0.27 nm and planes (102, typical of graphitic carbon). Various analytical techniques, including UV–vis spectroscopy, fluorescence spectroscopy, and TEM, were used to characterize the carbon-based nanomaterials, confirming their uniform size distribution (1.8 ± 2 nm), high FQY of 46.4%, and strong blue fluorescence due to Eu^3+^ doping. Beyond their Hg^2+^ sensing capabilities, the Eu^3+^ hybrid CDs were successfully used for the imaging of fungal cells, namely *Fomitopsis* sp., exhibiting multicolour emissions (blue, green, and red) across the cell membrane and successfully penetrate intracellular regions, demonstrating their effective internalization within the cells.

Nontoxic, blue emitting Eu^3+^ hybrid CDs were also used as fluorescent imaging agents to track three pathogenic *Candida* species: *C. albicans*, *C. parapsilosis*, and *C. tropicalis*.^145^ In this study, CDs were hydrothermally synthesized from histidine as the carbon source, APTES as the silicon source, and Eu­(NO_3_)_3_ as the metal dopant. When Eu^3+^ hybrid CDs were incubated with *Candida* species, only *C. tropicalis* showed weak fluorescence signals, regardless of the longer incubation time. In contrast, *C. albicans* and *C. parapsilosis* showed strong fluorescence after 60 min of staining. The variation in staining efficiency was attributed to the interactions between the hydrophilic nature of the Eu^3+^ hybrid CDs (featuring −NH_2_, −OH, and −COOH groups on the surface) and the hydrophilic/hydrophobic surface characteristics of the *Candida* species. The more hydrophilic surface of *C. tropicalis* allowed faster internalization of the Eu^3+^ hybrid CDs, whereas *C. albicans* and *C. parapsilosis* required longer incubation times for the Eu^3+^ hybrid CDs to penetrate the cell membranes. Additionally, Eu^3+^ hybrid CDs could effectively discriminate between live and dead *Candida* species based on the localization and intensity of fluorescence signals as well as the staining time. In fact, Eu^3+^ hybrid CDs could effectively stain yeast cells of all three dead species. The fluorescence signals were more uneven in the dead cells, likely due to differences in the substances that interact with the Eu^3+^ hybrid CDs. Notably, *C. parapsilosis* cells exhibited the brightest fluorescence, suggesting that the Eu^3+^ hybrid CDs could penetrate most yeast cells of dead Candida species via passive diffusion, particularly when the cell surface was damaged.

In the context of sensing applications, Gaikwad et al.^146^ developed a fluorescent sensor for fungal spore detection using CDs synthesized from citric acid through a combustion process at a liquid–liquid interface. A layer of petrol at the interface of an aqueous citric acid solution was ignited to trigger thermal decomposition of citric acid. The purified CDs were deposited as a thin layer on a quartz plates. The quenching of CD fluorescence upon interaction with fungal spores served as a quantification parameter, enabling fungal detection with a sensitivity as low as 0.93 μg/mL for *A. niger* spores. The novel sensors were used to measure the presence of common environmental fungal spores such as *A. niger*, *P. chrysogenum*, and *A. alternata*.

## 6 Conclusions and Future Perspectives

CDs have emerged as highly promising nanomaterials, offering a wide range of functional applications due to their unique physicochemical properties, including tunable fluorescence, high biocompatibility, and excellent antimicrobial activity. The use of CDs in antifungal applications offers a transformative strategy to combat fungal infections, tackling the challenges of drug resistance and the limited therapeutic efficacy of current treatments.

Since the initial discovery of CDs, many different synthetic approaches have been reported for their preparation. However, as we discussed in the introduction of this review, small changes in the reaction protocol and starting materials have a tremendous effect on the final molecular composition and structure of the CDs and in turn their properties and functional applications.

Custom synthesis and surface modifications have been developed to further enhance the potency and targeting ability of these materials, as evidenced by the successful *in vivo* and *in vitro* studies that we have discussed. These advancements position CDs and their composite materials as promising tunable nanomaterials for safe, sustainable, and innovative antifungal solutions across various fields. CDs generated from small molecules or sustainable sources have provided a plethora of functionality that has been exploited for bioimaging applications where live vs dead cells can be differentiated by their different interactions with CDs, for the selective targeting of specific microbial species, and for antifungal and biofilm inhibition applications. The ability to promote ROS generation through photodynamic effects can be used to disrupt fungal cell membranes, positioning these carbon-based nanomaterials as potent antifungal agents. In the medical sector, CDs have shown potential as antifungal drug delivery vectors, pathogen detection, and fluorescent probes for imaging and diagnostic purposes. Their use as antifungal agents extends beyond treating microbial infections to applications in tissue engineering, wound healing, and even as protective coatings in medical devices to prevent fungal biofilm formation. Outside the medical field, the versatility of CDs enables their incorporation into functional materials, such as food packaging films, textiles, and agricultural coatings. These applications contribute to enhanced food preservation, improved crop protection, and environmental sustainability. The use of sustainable and renewable sources for the synthesis of CDs, has paved the way for eco-friendly solutions that reduce reliance on toxic chemicals, positioning these materials as key components in the development of next-generation materials with antimicrobial and multifunctional capabilities. Their potential to revolutionize various industries, from healthcare to agriculture and food packaging, underscores the need for further exploration and innovation of CD-based technologies and the development of robust synthetic protocols and characterization strategies to ensure batch to batch material reproducibility.

## Vocabulary

Nanomaterials: A material that has at least one external dimension in the nanoscale range, typically between 1 and 100 nm. Nanomaterials often exhibit unique physical, chemical, mechanical, or optical properties that differ significantly from their bulk counterparts. These properties are attributed to the high surface area-to-volume ratio and quantum effects, making nanomaterials valuable in a wide range of fields, including medicine, electronics, energy, and environmental science.
Carbon dots (CDs): A class of fluorescent carbon-based nanomaterials typically less than 10 nm in size, which feature an amorphous or crystalline core and decorated with different functional group on the surface, e.g., OH, amine, carbohylic groups. These nanomaterials exhibit unique optical properties, especially strong and tunable photoluminescence, and are known for their water solubility, chemical stability, low toxicity, and biocompatibility. These properties make carbon dots highly suitable for applications in bioimaging, sensing, drug delivery, and optoelectronics.
Nanocomposites: A multiphase material composed of a matrix (such as a polymer, metal, or ceramic) embedded with nanomaterials to significantly enhance the material’s properties. Typical nanoscale additives such as carbon nanotubes, carbon dots, graphene, clay nanoparticles, or metal oxides, can improve mechanical strength, thermal stability, electrical conductivity, barrier properties, or chemical resistance of the base material. Nanocomposites are widely used in fields such as aerospace, automotive, electronics, packaging, and biomedicine due to their ability to combine lightweight structure with superior performance.
Fungi: Fungi are a kingdom of eukaryotic organisms that include microorganisms such as yeasts, molds, and mushrooms. They are characterized by having cell walls made of chitin, absorbing nutrients from organic material through external digestion, and reproducing via spores. Fungi can be unicellular (like yeasts) or multicellular (like molds and mushrooms), and they play essential roles in ecosystems as decomposers, symbionts, and pathogens. Unlike plants, fungi do not perform photosynthesis.
Antifungal agents: Substances used in medicine or agricultural to prevent, control, or eliminate fungal infections or infestations. These treatments typically inhibit the growth of or kill fungi. The goal of fungal treatments is to reduce harm caused by fungi while minimizing resistance and side effects.

## References

[1] Sreenivasan V. K., Zvyagin A. V., Goldys E. M. (2013). Luminescent
nanoparticles and their applications in the life sciences. J. Phys.: Condens. Matter.

[2] Liu J., Li R., Yang B. (2020). Carbon Dots:
A New Type of Carbon-Based Nanomaterial
with Wide Applications. ACS Cent Sci..

[3] Garcia-Millan T., Ramos-Soriano J., Ghirardello M., Liu X., Santi C. M., Eloi J. C., Pridmore N., Harniman R. L., Morgan D. J., Hughes S. (2023). Multicolor Photoluminescent Carbon Dots a La Carte
for Biomedical
Applications. ACS Appl. Mater. Interfaces.

[4] Li Q., Ohulchanskyy T. Y., Liu R., Koynov K., Wu D., Best A., Kumar R., Bonoiu A., Prasad P. N. (2010). Photoluminescent
carbon dots as biocompatible nanoprobes for targeting cancer cells
in vitro. J. Phys. Chem. C.

[5] Hill S., Galan M. C. (2017). Fluorescent carbon
dots from mono- and polysaccharides:
synthesis, properties and applications. Beilstein
J. Org. Chem..

[6] Li H., Huang J., Song Y., Zhang M., Wang H., Lu F., Huang H., Liu Y., Dai X., Gu Z. (2018). Degradable Carbon Dots
with Broad-Spectrum Antibacterial Activity. ACS Appl. Mater. Interfaces.

[7] Lim S. Y., Shen W., Gao Z. (2015). Carbon quantum
dots
and their applications. Chem. Soc. Rev..

[8] Đorđevic L., Arcudi F., Cacioppo M., Prato M. (2022). A multifunctional chemical
toolbox to engineer carbon dots for biomedical and energy applications. Nat. Nanotechnol.

[9] Xu X. Y., Ray R., Gu Y. L., Ploehn H. J., Gearheart L., Raker K., Scrivens W. A. (2004). Electrophoretic
analysis and purification
of fluorescent single-walled carbon nanotube fragments. J. Am. Chem. Soc..

[10] Zheng X. T., Ananthanarayanan A., Luo K. Q., Chen P. (2015). Glowing Graphene Quantum
Dots and Carbon Dots: Properties, Syntheses, and Biological Applications. Small.

[11] Kang Z., Lee S.-T. (2019). Carbon dots: advances
in nanocarbon applications. Nanoscale.

[12] Meng W., Bai X., Wang B., Liu Z., Lu S., Yang B. (2019). Biomass-Derived Carbon Dots and Their
Applications. Energy Environ. Mat.

[13] Baker S. N., Baker G. A. (2010). Luminescent Carbon
Nanodots: Emergent Nanolights. Angew. Chem.
Int. Edit.

[14] Ozyurt D., Kobaisi M. A., Hocking R. K., Fox B. (2023). Properties, synthesis,
and applications of carbon dots: A review. Carbon
Trends.

[15] Bruno F., Sciortino A., Buscarino G., Soriano M. L., Ríos Á., Cannas M., Gelardi F., Messina F., Agnello S. (2021). A Comparative
Study of Top-Down and Bottom-Up Carbon Nanodots and Their Interaction
with Mercury Ions. Nanomaterials.

[16] Garcia-Millan T., Swift T. A., Morgan D. J., Harniman R. L., Masheder B., Hughes S., Davis S. A., Oliver T. A. A., Galan M. C. (2022). Small variations
in reaction conditions tune carbon dot fluorescence. Nanoscale.

[17] Szczepankowska J., Khachatryan G., Khachatryan K., Krystyjan M. (2023). Carbon Dots—Types,
Obtaining and Application in Biotechnology and Food Technology. Int. J. Mol. Sci..

[18] Liu H., Zhong X., Pan Q., Zhang Y., Deng W., Zou G., Hou H., Ji X. (2024). A review of carbon dots in synthesis strategy. Coord. Chem. Rev..

[19] Ai L., Yang Y., Wang B., Chang J., Tang Z., Yang B., Lu S. (2021). Insights into
photoluminescence mechanisms
of carbon dots: advances and perspectives. Science
Bulletin.

[20] Tao S., Song Y., Zhu S., Shao J., Yang B. (2017). A new type of polymer carbon dots
with high quantum yield: From synthesis to investigation on fluorescence
mechanism. Polymer.

[21] Liu M. L., Chen B. B., Li C. M., Huang C. Z. (2019). Carbon dots: synthesis,
formation mechanism, fluorescence origin and sensing applications. Curr. Green Chem..

[22] Swift T. A., Duchi M., Hill S. A., Benito-Alifonso D., Harniman R. L., Sheikh S., Davis S. A., Seddon A. M., Whitney H. M., Galan M. C. (2018). Surface
functionalisation
significantly changes the physical and electronic properties of carbon
nano-dots. Nanoscale.

[23] Chen Y., Lian H., Wei Y., He X., Chen Y., Wang B., Zeng Q., Lin J. (2018). Concentration-induced
multi-colored emissions in carbon dots: origination from triple fluorescent
centers. Nanoscale.

[24] Li D., Jing P., Sun L., An Y., Shan X., Lu X., Zhou D., Han D., Shen D., Zhai Y. (2018). Near-Infrared Excitation/Emission
and Multiphoton-Induced Fluorescence
of Carbon Dots. Adv. Mater..

[25] Wang Y., Hu A. (2014). Carbon quantum dots:
synthesis, properties and applications. J. Mater.
Chem. C.

[26] Hola K., Sudolska M., Kalytchuk S., Nachtigallova D., Rogach A. L., Otyepka M., Zboril R. (2017). Graphitic Nitrogen
Triggers Red Fluorescence in Carbon Dots. ACS
Nano.

[27] Dhenadhayalan N., Lin K.-C., Suresh R., Ramamurthy P. (2016). Unravelling
the Multiple Emissive States in Citric-Acid-Derived Carbon Dots. J. Phys. Chem. C.

[28] Yan F., Sun Z., Zhang H., Sun X., Jiang Y., Bai Z. (2019). The fluorescence mechanism of carbon
dots, and methods for tuning their emission color: a review. Microchim Acta.

[29] Li W., Wei Z., Wang B., Liu Y., Song H., Tang Z., Yang B., Lu S. (2020). Carbon quantum
dots enhanced the
activity for the hydrogen evolution reaction in ruthenium-based electrocatalysts. Mater. Chem. Front..

[30] Sun X., Lei Y. (2017). Fluorescent carbon
dots and their sensing applications. Trends
Analyt Chem..

[31] Ghirardello M., Ramos-Soriano J., Galan M. C. (2021). Carbon Dots as an Emergent Class
of Antimicrobial Agents. Nanomaterials.

[32] Sturabotti E., Camilli A., Leonelli F., Vetica F. (2024). Carbon Dots as Bioactive
Antifungal Nanomaterials. ChemMedChem..

[33] Innocenzi P., De Forni D., Lori F. (2025). Antiviral Activity of Carbon Dots:
Strategies and Mechanisms of Action. Small Struct.

[34] Xu X., Ray R., Gu Y., Ploehn H. J., Gearheart L., Raker K., Scrivens W. A. (2004). Electrophoretic
analysis and purification of fluorescent single-walled carbon nanotube
fragments. J. Am. Chem. Soc..

[35] Mehta V. N., Jha S., Singhal R. K., Kailasa S. K. (2014). Preparation of multicolor emitting carbon dots for
HeLa cell imaging. New J. Chem..

[36] Samphire J., Takebayashi Y., Hill S. A., Hill N., Heesom K. J., Lewis P. A., Alibhai D., Bragginton E. C., Dorh J., Dorh N. (2022). Green fluorescent carbon
dots as targeting probes for LED-dependent
bacterial killing. Nano Select.

[37] Swift T. A., Fagan D., Benito-Alifonso D., Hill S. A., Yallop M. L., Oliver T. A. A., Lawson T., Galan M. C., Whitney H. M. (2021). Photosynthesis and crop productivity
are enhanced by glucose-functionalised carbon dots. New Phytol..

[38] Zhao L., Zhang M., Mujumdar A. S., Wang H. (2023). Application of carbon dots in food preservation: a critical review
for packaging enhancers and food preservatives. Crit Rev. Food Sci. Nutri.

[39] Ramos-Soriano J., Ghirardello M., Galan M. C. (2022). Carbon-based glyco-nanoplatforms:
towards the next generation of glycan-based multivalent probes. Chem. Soc. Rev..

[40] Hutton G. A.
M., Martindale B. C. M., Reisner E. (2017). Carbon dots as photosensitisers for solar-driven catalysis. Chem. Soc. Rev..

[41] Kang Z., Lee S. T. (2019). Carbon dots: advances in nanocarbon applications. Nanoscale.

[42] Molaei M. J. (2019). Carbon quantum dots and their biomedical
and therapeutic applications: a review. RSC
Adv..

[43] Fisher M. C., Alastruey-Izquierdo A., Berman J., Bicanic T., Bignell E. M., Bowyer P., Bromley M., Bruggemann R., Garber G., Cornely O. A. (2022). Tackling the emerging
threat of antifungal resistance to human health. Nat. Rev. Microbiol.

[44] Erwig L. P., Gow N. A. R. (2016). Interactions of fungal pathogens
with phagocytes. Nature Rev. Microbiol.

[45] Armstrong-James D., Meintjes G., Brown G. D. (2014). A neglected epidemic: fungal infections
in HIV/AIDS. Trends in Microbiology.

[46] European
Food and Safety Authority (2025). Impact of the use of azole fungicides, other than as human medicines,
on the development of azole-resistant Aspergillus spp. EFSA.

[47] Bottery M. J., van Rhijn N., Chown H., Rhodes J. L., Celia-Sanchez B. N., Brewer M. T., Momany M., Fisher M. C., Knight C. G., Bromley M. J. (2024). Elevated mutation rates in multi-azole resistant Aspergillus
fumigatus drive rapid evolution of antifungal resistance. Nat. Commun..

[48] Knoll M. A., Steixner S., Lass-Flörl C. (2023). How to use
direct microscopy for
diagnosing fungal infections. Clin Microbiol
Infec.

[49] Gow N. A. R., Latge J.-P., Munro C. A. (2017). The Fungal Cell
Wall: Structure,
Biosynthesis, and Function. Microbiology Spectrum.

[50] Pan X., Zhang Y., Sun X., Pan W., Yu G., Zhao Q., Wang J. (2018). Carbon dots originated
from methyl red with molecular state and surface state controlled
emissions for sensing and imaging. J. Luminesc.

[51] Tian B., Fu T., Wan Y., Ma Y., Wang Y., Feng Z., Jiang Z. (2021). B- and N-doped carbon dots by one-step microwave hydrothermal synthesis:
tracking yeast status and imaging mechanism. J. Nanobiotech.

[52] Rais A., Sharma S., Mishra P., Khan L. A., Prasad T. (2024). Biocompatible
carbon quantum dots as versatile imaging nanotrackers of fungal pathogen
– Candida albicans. Nanomed.

[53] Dong R., Li W., Kang Y., Yang X., Qu S., Zhang X., Zhang H., Zheng M., Zheng Y., Yang Q. (2021). Uptake, translocation
and toxicity of fluorescent carbon dots in oyster mushroom (Pleurotus
ostreatus). J. Lumin..

[54] Färkkilä S. M. A., Mortimer M., Jaaniso R., Kahru A., Kiisk V., Kikas A., Kozlova J., Kurvet I., Mäeorg U., Otsus M. (2024). Comparison
of Toxicity and Cellular Uptake of CdSe/ZnS
and Carbon Quantum Dots for Molecular Tracking Using Saccharomyces
cerevisiae as a Fungal Model. Nanomaterials.

[55] Chen J., Liu W. R., Li Y., Zou X., Li W., Liang J., Zhang H., Liu Y., Zhang X., Hu C. (2022). Architecting ultra-bright silanized
carbon dots by alleviating the
spin-orbit coupling effect: a specific fluorescent nanoprobe to label
dead cells. Chem. Engin J..

[56] Zhang Y., Liu K., Yu J., Chen H., Fu R., Zhu S., Chen Z., Wang S., Lu S. (2022). Single stain
hyperspectral
imaging for accurate fungal pathogens identification and quantification. Nano Res..

[57] Li M., Yang B., Tang J., Ning M., Guan Z., Li Z., Ye B., Zhong H., Guo Z., Liu Z. (2024). Dynamic visualization
monitoring of cell membrane damage using polarity-responsive amphiphilic
carbon dots. Chem. Engin J..

[58] Nong S., Wang M., Wang X., Li Y., Yu S., Tang C., Li G., Xu L. (2024). A multifunctional guanosine-based
carbon dots for dead microbial imaging and synergistic broad-spectrum
antimicrobial therapy. Chem. Engin J..

[59] Yu X. W., Liu X., Jiang Y. W., Li Y. H., Gao G., Zhu Y. X., Lin F., Wu F. G. (2022). Rose Bengal-Derived Ultrabright Sulfur-Doped Carbon
Dots for Fast Discrimination between Live and Dead Cells. Anal. Chem..

[60] Wang Z., Xu K.-F., Wang G., Durrani S., Lin F., Wu F.-G. (2023). “One stone,
five birds”: Ultrabright and multifaceted
carbon dots for precise cell imaging and glutathione detection. Chem. Engin J..

[61] Zhao D., Liu X., Zhang R., Huang X., Xiao X. (2021). Facile one-pot synthesis of multifunctional
protamine sulfate-derived carbon dots for antibacterial applications
and fluorescence imaging of bacteria. New J.
Chem..

[62] Jin X., Sun X., Chen G., Ding L., Li Y., Liu Z., Wang Z., Pan W., Hu C., Wang J. (2015). PH-sensitive
carbon dots for the visualization of regulation of intracellular pH
inside living pathogenic fungal cells. Carbon.

[63] Sun M.-Y., Tian B.-H., Li X.-X., Li Y., Lei Y., Guo X.-L., Miao Q., Li H., Liang H.-X. (2024). Carbon
dots with tunable excitation-independent fluorescence and organelle-specific
targeting via core graphitization and surface groups engineering. Chem. Engin J..

[64] Wang X., Wang Y., Pan W., Wang J., Sun X. (2021). Carbon-Dot-Based
Probe Designed to Detect Intracellular pH in Fungal Cells for Building
Its Relationship with Intracellular Polysaccharide. ACS Sust Chem. Engin.

[65] Zhang Y., Zhao J., Sun X., Pan W., Yu G., Wang J. (2018). Fluorescent carbon dots for probing
the effect of thiram on the membrane
of fungal cell and its quantitative detection in aqueous solution. Sens Actuators, B: Chem..

[66] Granados J.
A. O., Thangarasu P., Singh N., Vázquez-Ramos J. M. (2019). Tetracycline and
its quantum dots for recognition of Al3+ and application in milk developing
cells bio-imaging. Food Chem..

[67] Chen X., Li W., Chen J., Zhang X., Zhang W., Duan X., Lei B., Huang R. (2022). Transcriptomics
Integrated with Metabolomics Reveals
2-Methoxy-1, 4-Naphthoquinone-Based Carbon Dots Induced Molecular
Shifts in Penicillium italicum. J. Fungi.

[68] Liu Y., Li W., Wu K., Lei B., Chen J., Zhang X., Lei H., Duan X., Huang R. (2023). Antifungal molecular details of MNQ-derived
novel carbon dots against Penicillium digitatum. Food Chem..

[69] Kostov K., Andonova-Lilova B., Smagghe G. (2022). Inhibitory activity of carbon quantum
dots against Phytophthora infestans and fungal plant pathogens and
their effect on dsRNA-induced gene silencing. Biotech Biotech Equip.

[70] Qie X., Zan M., Li L., Gui P., Chang Z., Ge M., Wang R. S., Guo Z., Dong W. F. (2020). High photoluminescence
nitrogen, phosphorus co-doped carbon nanodots for assessment of microbial
viability. Coll Surf. B: Biointerf.

[71] Yin J., Zhao J., Wang Z., Xue F., Wang Q., Guo H., Cheng H., Li J., Shen J., Yin M. (2024). Preparation
of salicylic acid nano-protectant with dual synergistic mechanism:
High direct fungicidal activity and plant defence toward cotton Verticillium
wilt. Chem. Engin J..

[72] Wang Z., Li Y., Zhang B., Gao X., Shi M., Zhang S., Zhong S., Zheng Y., Liu X. (2023). Functionalized
Carbon
Dot-Delivered RNA Nano Fungicides as Superior Tools to Control Phytophthora
Pathogens through Plant RdRP1Mediated Spray-Induced Gene Silencing. Adv. Funct Mat.

[73] Gyawali B., Rahimi R., Alizadeh H., Mohammadi M. (2024). Graphene Quantum
Dots (GQD)-Mediated dsRNA Delivery for the Control of Fusarium Head
Blight Disease in Wheat. ACS Appl. Bio Mat.

[74] Fanning S., Mitchell A. P. (2012). Fungal biofilms. PLoS Pathog.

[75] Sturabotti E., Camilli A., Georgian
Moldoveanu V., Bonincontro G., Simonetti G., Valletta A., Serangeli I., Miranda E., Amato F., Giacomo Marrani A. (2024). Targeting
the Antifungal Activity of Carbon Dots against Candida albicans Biofilm
Formation by Tailoring Their Surface Functional Groups. Chem.—Eur. J..

[76] Li X., Huang R., Tang F. K., Li W. C., Wong S. S. W., Leung K. C. F., Jin L. (2019). Red-Emissive Guanylated Polyene-Functionalized
Carbon Dots Arm Oral Epithelia against Invasive Fungal Infections. ACS Appl. Mat Interf.

[77] Bagheri Z., Ehtesabi H., Hallaji Z., Aminoroaya N., Tavana H., Behroodi E., Rahimifard M., Abdollahi M., Latifi H. (2018). On-chip analysis of carbon dots effect
on yeast replicative lifespan. Anal Chim Act.

[78] Gao Z., Li X., Shi L., Yang Y. (2019). Deep eutectic solvents-derived carbon
dots for detection of mercury (II), photocatalytic antifungal activity
and fluorescent labeling for C. albicans. Spectrochim
Act Part A: Mol. Biomol Spect.

[79] Li X., Wu X., Yuan T., Zhu J., Yang Y. (2021). Influence of the iodine
content of nitrogen- and iodine-doped carbon dots as a peroxidase
mimetic nanozyme exhibiting antifungal activity against C. albicans. Biochem Engin J..

[80] Yan C., Wang C., Shao X., Shu Q., Hu X., Guan P., Teng Y., Cheng Y. (2021). Dual-targeted
carbon-dot-drugs nanoassemblies for modulating Alzheimer’s
related amyloid-β aggregation and inhibiting fungal infection. MatToday Bio.

[81] Song W., Wang X., Nong S., Wang M., Kang S., Wang F., Xu L. (2024). D-cysteine-Derived
Carbon Dots for
Selective Discrimination, Imaging, and Synergistic Elimination of
Gram-Positive Bacteria and Fungi. Adv. Funct
Mat.

[82] Belal A., Almalki A. H., Farghali A. A., Mahmoud R., Atta R. R., Allah A. E., Hassan W. H., Lee S., Kotp A. A., Essam D. (2024). Nitrogen-doped carbon quantum dots as a novel treatment
for black fungal bone infections (Mucormycosis): in vitro and in vivo
study. Artificial Cells, Nanomed Biotechnol.

[83] Chen H., Geng X., Ning Q., Shi L., Zhang N., He S., Zhao M., Zhang J., Li Z., Shi J. (2024). Biophilic Positive Carbon Dot Exerts Antifungal Activity and Augments
Corneal Permeation for Fungal Keratitis. Nano
Lett..

[84] Kansay V., Sharma V. D., Chandan G., Sharma I., Chakrabarti S., Bera M. K. (2023). Sustainable synthesis
of nitrogen-doped fluorescent carbon quantum dots derived from Cissus
quadrangularis for biomarker applications. Mater.
Chem. Phys..

[85] Gedda G., Sankaranarayanan S. A., Putta C. L., Gudimella K. K., Rengan A. K., Girma W. M. (2023). Green synthesis of multi-functional
carbon dots from medicinal plant leaves for antimicrobial, antioxidant,
and bioimaging applications. Sci. Rep.

[86] Rimal V., Shishodia S., Srivastava P. K., Gupta S., Mallick A. I. (2021). Synthesis
and characterization of Indian essential oil Carbon Dots for interdisciplinary
applications. Appl. Nanosci.

[87] Khan B., Zhang J., Durrani S., Wang H., Nawaz A., Durrani F., Ye Y., Wu F.-G., Lin F. (2024). Carbon-Dots-Mediated
Improvement of Antimicrobial Activity of Natural Products. ACS Appl. Mater. Interfaces.

[88] Jhonsi M. A., Ananth D. A., Nambirajan G., Sivasudha T., Yamini R., Bera S., Kathiravan A. (2018). Antimicrobial
activity, cytotoxicity and DNA binding studies of carbon dots. Spectrochimica Acta Part A: Molecular and Biomolecular Spectroscopy.

[89] Asha
Jhonsi M., Thulasi S. (2016). A novel fluorescent carbon dots derived
from tamarind. Chem. Phys. Lett..

[90] Zhao S., Huang L., Xie Y., Wang B., Wang F., Lan M. (2021). Green synthesis of
multifunctional carbon dots for anti-cancer and
anti-fungal applications. Chin J. Chem. Engin.

[91] Muktha H., Sharath R., Kottam N., Smrithi S. P., Samrat K., Ankitha P. (2020). Green Synthesis of
Carbon Dots and Evaluation of Its
Pharmacological Activities. BioNanoSci..

[92] Sheikh M. A., Chandok R. S., Abida K. (2023). High energy density storage, antifungal
activity and enhanced bioimaging by green self-doped heteroatom carbon
dots. Discover Nano.

[93] Zhao X., Wang L., Ren S., Hu Z., Wang Y. (2021). One-pot synthesis
of Forsythia@carbon quantum dots with natural anti-wood rot fungus
activity. Mat Design.

[94] Wang L., Wang T., Hao R., Wang Y. (2024). Construction
Strategy
and Mechanism of a Novel Wood Preservative with Excellent Antifungal
Effects. Molecules.

[95] Mogharbel A. T., Abu-Melha S., Hameed A., M. S. Attar R., Alrefaei A. F., Almahri A., El-Metwaly N. (2023). Anticancer
and microbicide action of carbon quantum dots derived from microcrystalline
cellulose: Hydrothermal versus infrared assisted techniques. Arab J. Chem..

[96] Thirumalaivasan N., Mahapatra S., Ramanathan G., Kumar A., Raja T., Muthuramamoorthy M., Pandit B., Pandiaraj S., Prakash S. (2024). Exploring antimicrobial and biocompatible applications
of eco-friendly fluorescent carbon dots derived from fast-food packaging
waste transformation. Environ. Res..

[97] Parveen S., Nazeer S., Chotana G. A., Kanwal A., Batool B., Bukhari N., Yaqoob A., Talib F. (2024). Designing of chitosan/gelatin
based nanocomposite films integrated with Vachellia nilotica gum carbon
dots for smart food packaging applications. Int. J. Biol. Macromol..

[98] Guo B., Liu G., Ye W., Xu Z., Li W., Zhuang J., Zhang X., Wang L., Lei B., Hu C. (2024). Multifunctional
carbon dots reinforced gelatin-based coating film for strawberry preservation. Food Hydrocoll.

[99] Ghorbani M., Tajik H., Moradi M., Molaei R., Alizadeh A. (2022). One-pot microbial
approach to synthesize carbon dots from baker’s yeast-derived
compounds for the preparation of antimicrobial membrane. J. Environ. Chem. Engin.

[100] Li Y., Yang J., Sun L., Liu B., Li H., Peng L. (2023). Crosslinked fish scale gelatin/alginate dialdehyde
functional films
incorporated with carbon dots derived from pomelo peel waste for active
food packaging. Int. J. Biol. Macromol..

[101] Sun X., Luo S., Zhang L., Miao Y., Yan G. (2024). Photodynamic
antibacterial activity of oxidase-like nanozyme based on long-lived
room-temperature phosphorescent carbon dots. Food Chem..

[102] Slewa L. H. (2024). Antifungal films for strawberry packaging
using carbon
quantum dots derived from lemon and onion juice via green hydrothermal
method. Food Bioscience.

[103] Kasibabu B. S. B., D’Souza S. L., Jha S., Singhal R. K., Basu H., Kailasa S. K. (2015). One-step synthesis
of fluorescent
carbon dots for imaging bacterial and fungal cells. Anal Methods.

[104] Kasibabu B. S. B., D’souza S. L., Jha S., Kailasa S. K. (2015). Imaging
of Bacterial and Fungal Cells Using Fluorescent Carbon Dots Prepared
from Carica papaya Juice. J. Fluoresc.

[105] Mehta V. N., Jha S., Basu H., Singhal R. K., Kailasa S. K. (2015). One-step hydrothermal
approach to fabricate carbon
dots from apple juice for imaging of mycobacterium and fungal cells. Sens Actuators B: Chem..

[106] Bhamore J. R., Jha S., Park T. J., Kailasa S. K. (2019). Green synthesis
of multi-color emissive carbon dots from Manilkara zapota fruits for
bioimaging of bacterial and fungal cells. Journal
of Photochem. Photobiol. B: Biol..

[107] Su Q., Gan L., Liu J., Yang X. (2020). Carbon dots derived
from pea for specifically binding with Cryptococcus neoformans. Anal. Biochem..

[108] Hua X.-W., Bao Y.-W., Wang H.-Y., Chen Z., Wu F.-G. (2017). Bacteria-derived
fluorescent carbon dots for microbial live/dead
differentiation. Nanoscale.

[109] Atchudan R., Edison T. N. J. I., Chakradhar D., Perumal S., Shim J.-J., Lee Y. R. (2017). Facile
green synthesis
of nitrogen-doped carbon dots using Chionanthus retusus fruit extract
and investigation of their suitability for metal ion sensing and biological
applications. Sens Actuators B: Chem..

[110] Patra S., Singh M., Subudhi S., Mandal M., Nayak A. K., Sahu B. B., Mahanandia P. (2023). One-step green
synthesis of in–situ functionalized carbon quantum dots from
Tagetes patula flowers: Applications as a fluorescent probe for detecting
Fe3+ ions and as an antifungal agent. Journal
of Photochem. Photobiol. A: Chem..

[111] Bhamore J. R., Jha S., Park T. J., Kailasa S. K. (2018). Fluorescence
sensing of Cu2+ ion and imaging of fungal cell by ultra-small fluorescent
carbon dots derived from Acacia concinna seeds. Sens. Actuators, B.

[112] Chaudhary S., Kumari M., Chauhan P., Ram Chaudhary G. (2021). Upcycling
of plastic waste into fluorescent carbon dots: An environmentally
viable transformation to biocompatible C-dots with potential prospective
in analytical applications. Waste Manag.

[113] Zheng X., Qin K., He L., Ding Y., Luo Q., Zhang C., Cui X., Tan Y., Li L., Wei Y. (2021). Novel fluorescent nitrogen-doped
carbon dots derived from Panax notoginseng
for bioimaging and high selectivity detection of Cr 6+. Analyst.

[114] Yu D., Wang L., Zhou H., Zhang X., Wang L., Qiao N. (2019). Fluorimetric Detection of Candida albicans Using Cornstalk N-Carbon
Quantum Dots Modified with Amphotericin B. Bioconjugate
Chem..

[115] Pandey P. K., Preeti, Rawat K., Prasad T., Bohidar H. B. (2020). Multifunctional,
fluorescent DNA-derived carbon dots for biomedical applications: bioimaging,
luminescent DNA hydrogels, and dopamine detection. J. Mater. Chem. B.

[116] Oliveira B. P. D., Bessa N. U. D. C., Do Nascimento J. F., De Paula Cavalcante C.
S., Fontenelle R. O. D. S., Abreu F. O. M. D. S. (2023). Synthesis of luminescent chitosan-based
carbon dots for Candida albicans bioimaging. Int. J. Biol. Macromol..

[117] Sharma N., Sharma I., Bera M. K. (2022). Microwave-Assisted
Green Synthesis of Carbon Quantum Dots Derived from Calotropis Gigantea
as a Fluorescent Probe for Bioimaging. J. Fluoresc.

[118] Purbia R., Paria S. (2016). A simple turn on fluorescent sensor
for the selective detection of thiamine using coconut water derived
luminescent carbon dots. Biosen Bioelectron.

[119] Liu W., Li C., Sun X., Pan W., Yu G., Wang J. (2017). Highly crystalline carbon dots from
fresh tomato: UV emission and
quantum confinement. Nanotechnology.

[120] Al-Anazi M. (2024). Gold versus platinum for chemical
modification of carbon
quantum dots from carboxymethyl cellulose: Tunable biomedical performance. Int. J. Biol. Macromol..

[121] Priyadarshini E., Rawat K., Prasad T., Bohidar H. B. (2018). Antifungal
efficacy of Au@ carbon dots nanoconjugates against opportunistic fungal
pathogen, Candida albicans. Coll Surf. B: Bioint.

[122] Li X., Xu Y., Ouyang D., Ye K., Chen Y., Li Q., Xia Q., Wu X., Yang Y. (2023). Copper- and iodine-doped
nanozymes with simulated enzyme activity and efficient antifungal
activity against Candida albicans. Biochem Engin
J..

[123] Zhao D., Liu X., Zhang R., Xiao X., Li J. (2021). Preparation of two types of silver-doped
fluorescent carbon dots
and determination of their antibacterial properties. J. Inorg. Biochem.

[124] Tenkayala N. K., Katari N. K., Gundla R., Jonnalagadda S. B., Devaraju S. (2024). Sustainable approach to synthesis of carbon Dot/ silver
nanoparticles for biological evaluation as antimicrobial agent. Mater. Res. Express.

[125] Slewa L. H., Gozeh B. A., Ismael D. S., FaqeAbdulla N. Q., Othman H. O. (2024). Antibacterial and Antifungal Activity of Ag-NPs Colloids
Prepared by a Hydrothermal Reaction in Green Synthesized CQD. BioNanoSci..

[126] Etefa H. F., Nemera D. J., Dejene F. B. (2023). Green Synthesis
of Nickel Oxide NPs Incorporating Carbon Dots for Antimicrobial Activities. ACS Omega.

[127] Zhang J., Liu S., Wang X., Yao J., Zhai M., Liu B., Liang C., Shi H. (2021). Highly efficient
Ti3+ self-doped TiO2 co-modified with carbon dots and palladium nanocomposites
for disinfection of bacterial and fungi. J.
Hazard Mat.

[128] Chen Y., Cheng H., Wang W., Jin Z., Liu Q., Yang H., Cao Y., Li W., Fakhri A., Gupta V. K. (2021). Preparation of carbon dots-hematite
quantum dots-loaded
hydroxypropyl cellulose-chitosan nanocomposites for drug delivery,
sunlight catalytic and antimicrobial application. Journal of Photochem. Photobiol. B: Biology.

[129] Chen S., Li X., Bai M., Shi S. Q., Aladejana J. T., Cao J., Li J. (2023). Oyster-inspired
carbon
dots-functionalized silica and dialdehyde chitosan to fabricate a
soy protein adhesive with high strength, mildew resistance, and long-term
water resistance. Carbohydr. Polym..

[130] Evseev Z. I., Tarasova L. A., Vasilieva F. D., Egorova M. N., Dmitriev P. S., Akhremenko Y. A., Smagulova S. A. (2024). Comparison of Antimicrobial Properties of Graphene
Oxide-Based Materials, Carbon Dots, and Their Combinations Deposited
on Cotton Fabrics. Int. J. Mol. Sci..

[131] Safitri I., Sugiarti S., Darmawan N. (2024). Carbon Dots-based
Antifungal
Coating Film Against Pathogens Colletotrichum sp. for Active Coating
Application of Mango. Sci. Technol. Indon.

[132] Ezati P., Rhim J.-W., Molaei R., Priyadarshi R., Han S. (2022). Cellulose nanofiber-based coating film integrated with nitrogen-functionalized
carbon dots for active packaging applications of fresh fruit. Postharvest Biol. Technol..

[133] Ezati P., Rhim J.-W. (2022). Pectin/carbon quantum
dots fluorescent
film with ultraviolet blocking property through light conversion. Coll Surf. B: Biointerf.

[134] Wang L., Zhao X., Ren S., Hu Z., Wang Y. (2023). Application
of pH-responsive functionalized hollow mesoporous organosilica
nanoparticles for wood preservation. Mat Design.

[135] Ezati P., Khan A., Priyadarshi R., Bhattacharya T., Tammina S. K., Rhim J.-W. (2023). Biopolymer-based
UV protection functional films for food packaging. Food Hydrocoll.

[136] Koshy R. R., Reghunadhan A., Mary S. K., Sadanandan S., Jose S., Thomas S., Pothen L. A. (2022). AgNP anchored carbon
dots and chitin nanowhisker embedded soy protein isolate films with
freshness preservation for active packaging. Food Packaging and Shelf Life.

[137] De B., Gupta K., Mandal M., Karak N. (2015). Biocide immobilized
OMMT-carbon dot reduced Cu2O nanohybrid/hyperbranched epoxy nanocomposites:
Mechanical, thermal, antimicrobial and optical properties. Mat Sci. Engin C.

[138] Alaş M. O., Dogan G., Yalcin M. S., Ozdemir S., Genç R. (2022). Multicolor Emitting Carbon Dot-Reinforced
PVA Composites
as Edible Food Packaging Films and Coatings with Antimicrobial and
UV-Blocking Properties. ACS Omega.

[139] Ezati P., Rhim J. W., Molaei R., Rezaei Z. (2022). Carbon quantum
dots-based antifungal coating film for active packaging application
of avocado. Food Packaging and Shelf Life.

[140] Ezati P., Rhim J. W., Molaei R., Priyadarshi R., Han S. (2022). Cellulose nanofiber-based coating
film integrated with nitrogen-functionalized
carbon dots for active packaging applications of fresh fruit. Postharvest Biol. Technol..

[141] Riahi Z., Rhim J. W., Bagheri R., Pircheraghi G., Lotfali E. (2022). Carboxymethyl cellulose-based functional
film integrated
with chitosan-based carbon quantum dots for active food packaging
applications. Progr Organ Coat..

[142] Riahi Z., Khan A., Rhim J. W., Shin G. H., Kim J. T. (2024). Synergistic effect of iron-based
metal-organic framework
hybridized with carbon quantum dots in agar/gelatin films for fruit
preservation. Food Pack Shelf Life.

[143] Khan A., Riahi Z., Kim J. T., Rhim J. W. (2024). Carboxymethyl
cellulose/gelatin film incorporated with eggplant peel waste-derived
carbon dots for active fruit packaging applications. Int. J. Biol. Macromol..

[144] Desai M. L., Jha S., Basu H., Singhal R. K., Sharma P. K., Kailasa S. K. (2018). Microwave-assisted
synthesis of water-soluble
Eu 3+ hybrid carbon dots with enhanced fluorescence for the sensing
of Hg 2+ ions and imaging of fungal cells. New
J. Chem..

[145] Liang C., Shi Q., Zhang Y., Xie X. (2023). Water-soluble
carbonized polymer dots with tunable solid- and dispersion-state fluorescence
for multicolor films, anti-counterfeiting, and fungal imaging. Mat Today Nano.

[146] Gaikwad A., Joshi M., Patil K., Sathaye S., Rode C. (2019). Fluorescent
Carbon-Dots Thin Film for Fungal Detection and Bio-labeling
Applications. ACS Applied Bio Mat.

